# Supporting dataset on the validation and verification of the analytical method for the biomonitoring of 360 toxicologically relevant pollutants in whole blood

**DOI:** 10.1016/j.dib.2020.105878

**Published:** 2020-06-18

**Authors:** Cristian Rial-Berriel, Andrea Acosta-Dacal, Fernando González, Natalia Pastor-Tiburón, Manuel Zumbado, Octavio P. Luzardo

**Affiliations:** a Toxicology Unit, Clinical Sciences Department, Research Institute of Biomedical and Health Sciences (IUIBS), Universidad de Las Palmas de Gran Canaria, Paseo Blas Cabrera Felipe s/n, 35016 Las Palmas de Gran Canaria, Spain; b Study Group on Wild Animal Conservation Medicine (GEMAS), Spain; c GREFA (Grupo de Rehabilitación de la Fauna Autóctona y su Hábitat), Ctra. Monte del Pilar s/n, 28220 Majadahonda, Madrid, Spain; d Spanish Biomedical Research Center in Physiopathology of Obesity and Nutrition (CIBERObn), Spain

**Keywords:** Persistent organic pollutants, Pesticides, Rodenticides, Veterinary pharmaceuticals, Barn owl, Common kestrel, GC-MS/MS, LC-MS/MS

## Abstract

The dataset presented in this article supports “Micro QuEChERS-based method for the simultaneous biomonitoring in whole blood of 360 toxicologically relevant pollutants for wildlife” (Rial-Berriel et al., 2020). The supplementary data are: (1) Detailed validation data of the LC-MS/MS and GC-MS/MS methods for the quantification of 360 chemicals covering bias and precision (intra- and inter-day variability) for retention times, linearity, and limits of quantification. (2) Graphical data of the matrix effects on the quantification of all of the analytes. (3) Individual data of the 51 chemicals detected in real whole blood samples from two raptor species: 36 barn owls (*Tyto alba*) and 112 common kestrels (*Falco tinnunculus*).

Specifications Table

**Table:** 

**Subject**	Environmental Chemistry
**Specific subject area**	Analytical chemistry applied to biological samples to perform biomonitoring of environmental pollutants
**Type of data**	Tables and figures
**How data were acquired**	Ultra-high performance liquid chromatography tandem coupled to triple quadrupole mass spectrometry (LC-MS/MS). Agilent Technologies (Palo Alto, USA) models 1290 (UHPLC) and 6460 (MS/MS). Gas chromatography tandem coupled to triple quadrupole mass spectrometry (GC-MS/MS). Agilent Technologies (Palo Alto, USA) models 7890B (GC) and 7010 (MS/MS).
**Data format**	Analyzed: • Extracted and analyzed LC-MS/MS and GC-MS/MS data for the validation studies • Quantified chromatogram data of pollutants in the blood of barn owls and common kestrels Raw data: • All raw data corresponding to the 5 replicates of each concentration tested in the validation experiments presented in Table 1. • All individual quantitative data obtained after application of the method on 148 common kestrels and 39 barn owls presented in Tables 2–5. • All raw data used for the elaboration of Fig. 1.
**Parameters for data collection**	The validation data of the developed method included, limit of quantification (as the lowest calibrator that fulfilled de validation criteria), linearity, accuracy (expressed as % bias and precision (intra- and inter-day RSDs)) for each of the 360 analytes. The matrix effect on the quantification of the analytes is graphically represented as percentages. For clarity the results are expressed as relative percentage. The quantification data were obtained analyzing a series of 148 blood samples, obtained from a field ecology work in nest boxes of barn owls (*Tyto alba, n* = 36), and common kestrels (*Falco tinnunculus, n* = 112).
**Description of data collection**	MassHunter Quantitative Analysis was employed to collect and analyze the chromatographic data delivered by the triple quadrupole mass spectrometers coupled to both UHPLC and GC. The linearity was assessed by injecting a 12-point calibration curve prepared in the blank matrix and extracted with the developed micro-QuEChERS method. To test bias and precision, standard solutions of 360 reference standards were employed to spike blank whole blood samples at five concentration levels (0.1, 0.5, 1, 5, and 20 ng/ml) were injected in quintuplicate. The bias and repeatability (intra-day variability) were determined by those quintuplicate analyses of each sample, as these were injected within 24 h. The reproducibility (inter-day variability) was measured on three non-consecutive days within a two-week span. The matrix effect was assessed by extracting enough amount of blank matrix with the developed method and fortifying these extracts with three levels of the mixture of 360 chemicals (0.2, 2, and 10 ng/ml), and quantified against a calibration curve prepared in the solvent (1% FA-acetonitrile). Regarding the quantitative data, the real samples were prepared with the developed methodology and analyzed by UHPLC and GC.
**Data source location**	Institution: Toxicology Unit, Research Institute of Biomedical and Health Sciences (IUIBS), University of Las Palmas de Gran Canaria City/Town/Region: Las Palmas de Gran Canaria Country: Spain
**Data accessibility**	With the article. Raw data are provided
**Related research article**	Rial-Berriel, C., Acosta-Dacal, A., Zumbado, M., Luzardo, O.P. Micro QuEChERS-based methodology for the simultaneous biomonitoring in whole blood of 360 toxicologically relevant pollutants for wildlife. Science of the Total Environment 736 (2020) 13944

## Value of the data

• An easy way to consult the validation data of the methodological development detailed in the main article is provided.
• Additionally, the validation data might serve as a reference to other researchers developing methods in complex biological matrices.
• The detail of the matrix effect on each of the 360 compounds analyzed may allow other researchers to decide whether they need to prepare their calibration lines in matrix or in solvent.
• Data on numerous environmental pollutants are presented for the first time for two apex predators: barn owl (*Tyto alba*) and kestrel (*Falco tinnunculus*).
• The biomonitoring data presented can be used by other researchers in ecotoxicology for comparison purposes, and for meta-analyses of chemical contamination in birds of prey.

## Data description

Table 1 shows the major validation parameters of each of the 360 chemicals optimized in this methodology. Validation criteria fulfilled those established in the SANTE guidelines [1]. Given the particularities of the matrix - whole blood - the guidelines of the SWGTOX were also considered, especially concerning the preparation of the matrix-matched calibration curve [2]. The data reported in this table complement those included in Table 1 of the article by Rial-Berriel et al. [3], where the parameters of identity and selectivity of each compound are shown. Now, in this table, the data of linearity (as a working range), the LOQ (set as the lowest point of the calibration curve that complied identity, bias and precision criteria), repeatability (as intraday RSD), and reproducibility (as interday RSD) are shown for each of the chemicals. The bias and precision (repeatability and reproducibility) data are presented for five fortification levels (0.1, 0.5, 1, 5, and 20 ng/ml). Raw data of the five replicates for each concentration are provided in the supplementary file 1.

**Table 1:** Results of validation process: LOQ, linearity, bias and precision (intraday and interday).

				**0.1** **ng/ml**			**0.5** **ng/ml**			**1** **ng/ml**			**5** **ng/ml**			**20** **ng/ml**		
					**Precision (RSD. %)**			**Precision (RSD. %)**			**Precision (RSD. %)**			**Precision (RSD. %)**			**Precision (RSD. %)**	
**N°**	**Compound**	**LOQ**	**Linearity**	**Bias (%)**	**Intraday**	**Interday**	**Rec. (%)**	**Intraday**	**Interday**	**Rec. (%)**	**Intraday**	**Interday**	**Rec. (%)**	**Intraday**	**Interday**	**Rec. (%)**	**Intraday**	**Interday**
1	2-Phenylphenol	0.20	0.9946	N/A	N/A	N/A	110.10	8,99	11.18	100.03	8,25	6.86	86.09	6,67	8.32	100.98	13,03	1.62
2	4.4′-Dichlorobenzophenone (metabolite of dicofol)	0.80	0.9905	N/A	N/A	N/A	N/A	N/A	N/A	100.35	8,41	12.21	101.69	6,90	17.71	96.39	13,85	1.72
3	Abamectine	4.00	0.9784	N/A	N/A	N/A	N/A	N/A	N/A	N/A	N/A	N/A	107.86	6,77	14.19	102.58	15,24	4.37
4	Acenaphthene	0.20	0.9889	N/A	N/A	N/A	83.21	17,09	8.12	124.25	9,64	7.02	94.92	6,78	10.11	96.71	7,51	8.70
5	Acenaphtylene	0.20	0.9792	N/A	N/A	N/A	108.10	14,25	15.63	99.81	10,39	5.64	94.84	15,98	10.11	102.65	7,26	9.23
6	Acephate	2.00	0.9879	N/A	N/A	N/A	N/A	N/A	N/A	N/A	N/A	N/A	119.99	13,67	0.16	103.25	7,44	12.14
7	Acetaminophen (paracetamol)	1.20	0.9548	N/A	N/A	N/A	N/A	N/A	N/A	126.11	7,73	13.96	79.54	17,10	13.04	105.72	14,92	29.30
8	Acetamiprid	0.40	0.9967	N/A	N/A	N/A	113.51	14,76	3.73	97.67	11,75	2.41	104.22	17,94	4.72	95.95	7,89	1.24
9	Acrinathrin	1.20	0.9943	N/A	N/A	N/A	N/A	N/A	N/A	109.46	9,36	10.87	93.81	6,62	5.87	99.70	16,60	9.75
10	Albendazole	0.10	0.9952	121.06	10,61	8.38	96.22	9,08	10.63	99.00	10,54	1.33	108.29	17,77	3.44	96.38	14,56	0.81
11	Aldicarb	0.10	0.9956	118.27	18,28	14.53	98.52	10,06	4.99	92.82	8,85	1.70	102.55	6,62	5.28	95.35	13,66	3.09
12	Aldicarb-sulfone	0.40	0.9966	N/A	N/A	N/A	118.79	12,30	5.58	93.35	10,83	7.71	99.82	6,62	4.56	96.53	7,31	1.87
13	Aldicarb-sulfoxide	1.60	0.9936	N/A	N/A	N/A	N/A	N/A	N/A	N/A	N/A	N/A	100.26	6,62	1.11	94.55	7,50	0.14
14	Aldrin	0.40	0.9951	N/A	N/A	N/A	95.34	15,89	4.08	97.07	13,35	9.52	95.19	6,86	0.34	97.66	7,25	1.76
15	Anthracene	0.80	0.9579	N/A	N/A	N/A	N/A	N/A	N/A	114.90	7,72	6.49	92.78	14,13	9.89	95.94	14,59	8.63
16	Atrazine	0.10	0.9960	107.95	8,69	1.74	97.69	16,36	17.22	93.12	13,51	3.34	103.37	15,25	5.44	95.62	8,06	0.71
17	Azinphos-methyl	0.20	0.9961	N/A	N/A	N/A	108.57	8,99	1.85	95.64	10,75	3.62	107.13	15,31	3.49	94.57	13,68	0.15
18	Azoxystrobin	0.10	0.9972	103.13	15,68	1.38	96.51	8,98	17.06	97.09	10,29	4.55	104.23	17,56	7.15	97.49	15,71	4.20
19	BDE-28	0.20	0.9932	N/A	N/A	N/A	103.43	8,92	12.34	101.66	8,32	5.74	90.52	6,73	9.65	90.60	17,40	8.15
20	BDE-47	0.20	0.9924	N/A	N/A	N/A	96.70	15,12	7.67	100.78	11,76	5.69	91.21	17,86	9.72	94.25	8,12	8.48
21	BDE-85	0.10	0.9954	129.76	10,28	5.15	91.22	13,95	11.00	101.98	12,44	5.76	86.00	7,35	9.16	99.65	7,39	8.96
22	BDE-99	0.10	0.9934	91.23	5,37	4.22	105.76	14,85	14.54	103.28	13,00	5.83	94.33	6,64	10.05	94.77	7,85	8.52
23	BDE-100	0.10	0.9873	109.21	8,67	7.98	89.95	10,40	11.20	101.34	10,20	5.72	92.07	6,78	9.81	85.36	16,69	7.68
24	BDE-153	0.20	0.9951	N/A	N/A	N/A	86.36	13,46	10.95	102.38	10,65	5.78	98.11	6,62	10.45	95.42	7,26	8.58
25	BDE-154	0.10	0.9954	111.23	7,50	8.20	109.41	9,31	11.22	101.80	8,92	5.75	94.84	15,34	10.11	91.95	15,43	8.27
26	BDE-183	0.20	0.9815	N/A	N/A	N/A	98.77	10,04	7.64	103.39	8,47	5.84	98.97	14,04	10.55	103.42	15,38	9.30
27	Benalaxyl	0.10	0.9978	97.07	10,61	4.88	91.75	10,74	5.04	96.40	9,17	0.54	102.02	17,93	2.34	98.23	14,16	2.16
28	Bendiocarb	0.10	0.9961	106.43	17,38	11.19	98.54	16,51	11.11	88.59	11,92	0.30	101.12	17,69	7.81	95.22	7,38	1.33
29	Bendiocarb metabolite (2. 2‐dimethylbenzo‐1. 3‐dioxol‐4‐ol)	1.20	0.9968	N/A	N/A	N/A	N/A	N/A	N/A	100.90	12,67	0.91	82.48	6,69	8.04	99.56	13,52	3.43
30	Benfuracarb	0.10	0.9950	86.06	8,05	10.19	103.65	14,71	15.73	99.20	13,07	2.80	106.49	13,63	4.43	95.72	14,04	5.01
31	Benzo[a]anthracene	0.80	0.9805	N/A	N/A	N/A	N/A	N/A	N/A	111.54	7,78	6.30	94.91	6,69	10.11	89.36	9,08	8.04
32	Benzo[a]pyrene	0.10	0.9948	124.45	14,20	9.32	95.76	14,39	6.53	112.23	10,04	6.34	95.02	6,67	10.13	101.13	12,65	9.09
33	Benzo[b]fluoranthene	0.80	0.9766	N/A	N/A	N/A	N/A	N/A	N/A	105.05	8,54	5.93	92.65	6,62	9.87	98.55	10,27	8.86
34	Benzo[ghi]perylene	0.40	0.9912	N/A	N/A	N/A	119.14	9,12	7.22	111.18	12,61	6.28	99.29	6,67	10.58	103.47	9,06	9.30
35	Benzo[k]fluoranthene	0.40	0.9323	N/A	N/A	N/A	95.12	9,17	9.12	95.35	15,14	5.39	100.05	16,54	10.66	94.17	10,62	8.47
36	Bifenthrin	0.20	0.9952	N/A	N/A	N/A	98.48	13,53	12.82	102.14	10,00	1.84	89.51	18,10	3.56	95.27	13,67	5.57
37	Bitertanol	0.40	0.9938	N/A	N/A	N/A	105.62	14,27	15.92	105.68	9,81	13.20	102.68	10,19	5.54	96.23	14,00	7.53
38	Boscalid (formerly nicobifen)	0.10	0.9974	112.80	9,55	7.39	101.63	14,86	4.10	97.54	10,08	6.17	84.80	10,06	7.47	100.58	12,54	5.73
39	Brodifacoum	0.80	0.9806	N/A	N/A	N/A	N/A	N/A	N/A	92.73	13,12	5.11	96.45	15,56	15.43	100.49	9,93	12.33
40	Bromadiolone	0.40	0.9864	N/A	N/A	N/A	116.06	10,17	18.62	89.90	9,56	5.46	112.93	9,46	3.98	100.80	16,46	0.11
41	Bromopropylate	0.20	0.9887	N/A	N/A	N/A	99.38	14,28	11.80	102.94	12,91	14.65	88.10	13,47	2.10	98.29	9,89	17.90
42	Bromuconazole (two isomers)	0.20	0.9821	N/A	N/A	N/A	97.91	12,92	8.33	98.52	13,61	11.16	87.58	14,54	19.50	99.45	9,70	9.08
43	Bupirimate	0.20	0.9940	N/A	N/A	N/A	97.15	13,95	10.65	97.41	12,47	1.67	88.40	9,36	2.98	97.21	10,00	5.20
44	Buprofezin	0.10	0.9930	111.89	12,12	7.55	93.70	10,78	4.48	91.32	8,97	3.26	96.80	11,53	3.61	97.21	15,68	7.98
45	Cadusafos (ebufos)	0.10	0.9956	86.62	9,70	9.96	94.38	9,34	11.50	103.53	9,81	5.16	105.85	13,64	4.40	95.85	15,07	2.52
46	Carbaryl	0.10	0.9978	106.07	9,25	5.31	94.48	9,87	3.35	98.38	10,04	9.69	105.00	13,67	4.09	96.64	14,97	3.48
47	Carbendazim (azole)	0.40	0.9965	N/A	N/A	N/A	118.80	13,28	3.68	94.83	13,87	3.95	101.66	13,34	4.74	97.47	9,11	2.27
48	Carbofuran	0.10	0.9959	118.09	12,71	9.84	93.02	11,68	15.57	90.26	9,61	3.87	98.83	12,60	9.04	96.21	14,66	4.67
49	Carbofuran-3-hydroxy	0.40	0.9963	N/A	N/A	N/A	117.69	12,90	6.37	96.99	13,89	4.38	103.10	13,92	4.90	95.70	9,44	2.86
50	Carbosulfan	0.40	0.9781	N/A	N/A	N/A	132.53	12,51	5.32	84.83	13,15	18.63	110.47	9,64	9.70	105.49	9,25	3.40
51	Cefuroxima axetil (two isomers)	0.80	0.9902	N/A	N/A	N/A	N/A	N/A	N/A	105.25	14,44	3.61	100.04	10,23	5.53	104.99	8,89	2.88
52	Chloramphenicol	2.00	0.9814	N/A	N/A	N/A	N/A	N/A	N/A	N/A	N/A	N/A	120.55	9,33	11.01	102.82	12,43	9.86
53	Chlorantraniliprole	0.20	0.9952	N/A	N/A	N/A	107.14	9,74	13.70	91.93	9,32	10.91	104.44	9,61	6.25	95.70	13,94	4.00
54	Chlorfenapyr	1.20	0.9936	N/A	N/A	N/A	N/A	N/A	N/A	97.07	8,89	12.36	85.57	13,88	11.55	106.01	14,26	1.61
55	Chlorfenvinphos	0.20	0.9969	N/A	N/A	N/A	96.71	14,12	11.93	100.05	14,95	5.73	102.06	9,37	0.48	96.97	9,73	0.29
56	Chlorobenzilate	0.40	0.9909	N/A	N/A	N/A	88.96	9,99	9.32	105.63	8,87	5.97	87.66	15,64	9.34	89.59	14,82	8.06
57	Chlorophacinone	0.80	0.9874	N/A	N/A	N/A	N/A	N/A	N/A	96.82	12,73	1.94	103.81	13,52	2.08	100.62	9,72	4.45
58	Chlorpropham	0.20	0.9949	N/A	N/A	N/A	110.12	13,64	10.89	101.16	13,17	9.22	85.27	16,60	9.82	97.24	10,40	9.23
59	Chlorpyrifos	0.80	0.9915	N/A	N/A	N/A	N/A	N/A	N/A	100.64	14,14	10.32	91.52	15,69	7.57	100.79	10,24	7.46
60	Chlorpyrifos methyl	0.40	0.9951	N/A	N/A	N/A	103.73	9,45	7.79	104.81	9,61	13.90	88.99	10,69	10.67	99.45	13,30	4.76
61	Chlorthal dimethyl	0.20	0.9874	N/A	N/A	N/A	91.40	11,74	4.48	107.91	8,89	7.07	90.69	9,27	3.38	96.20	14,69	7.87
62	Chrysene	0.80	0.9789	N/A	N/A	N/A	N/A	N/A	N/A	109.29	9,22	6.17	93.19	9,29	9.93	94.31	13,57	8.48
63	Clindamycin	0.40	0.9970	N/A	N/A	N/A	116.37	11,77	4.52	96.24	12,64	9.31	104.48	10,53	6.42	97.59	9,89	1.83
64	Clofentezine	0.40	0.9944	N/A	N/A	N/A	101.79	9,49	2.24	96.83	9,70	1.32	107.77	13,16	5.27	98.86	14,77	0.82
65	Clothianidin	1.20	0.9941	N/A	N/A	N/A	N/A	N/A	N/A	89.69	13,57	2.55	100.83	9,18	9.29	93.83	9,88	2.69
66	Cloxacillin	1.60	0.9803	N/A	N/A	N/A	N/A	N/A	N/A	N/A	N/A	N/A	110.09	14,70	3.00	101.78	9,10	3.38
67	Cortiscosterone 21 acetate	0.80	0.9948	N/A	N/A	N/A	N/A	N/A	N/A	116.41	14,25	25.41	105.76	12,62	1.63	97.81	9,06	4.02
68	Coumachlor	0.20	0.9948	N/A	N/A	N/A	95.39	9,51	2.10	101.51	9,31	2.76	109.44	14,42	6.63	99.44	16,00	6.43
69	Coumaphos	0.10	0.9977	116.47	12,99	9.03	87.29	10,10	2.13	93.40	8,98	5.88	102.10	12,07	4.65	98.22	13,49	5.08
70	Coumatetralyl	0.40	0.9791	N/A	N/A	N/A	104.44	9,23	14.38	104.06	9,03	13.27	91.59	9,96	1.15	98.21	14,37	11.04
71	Cyazofamid	0.80	0.9973	N/A	N/A	N/A	N/A	N/A	N/A	106.76	12,52	2.68	103.61	10,04	6.11	98.92	9,10	1.86
72	Cyflufenamid	0.20	0.9936	N/A	N/A	N/A	85.26	11,55	2.59	112.30	9,20	13.66	110.04	9,40	2.12	96.14	14,03	7.41
73	Cyfluthrin (sum of four isomers)	1.20	0.9923	N/A	N/A	N/A	N/A	N/A	N/A	102.22	15,22	2.03	89.47	10,43	0.08	100.93	9,55	0.81
74	Cyhalothrin (lambda isomer)	2.00	0.9938	N/A	N/A	N/A	N/A	N/A	N/A	N/A	N/A	N/A	95.52	15,51	10.00	100.53	9,65	1.89
75	Cymoxanil	0.40	0.9975	N/A	N/A	N/A	114.66	12,65	10.00	96.84	14,55	1.40	101.03	9,71	5.35	96.68	10,08	1.83
76	Cypermethrin (sum of four isomers)	4.00	0.9886	N/A	N/A	N/A	N/A	N/A	N/A	N/A	N/A	N/A	94.85	13,60	7.83	102.11	14,45	7.39
77	Cyproconazole (two isomers)	0.40	0.9952	N/A	N/A	N/A	102.85	10,03	18.32	98.08	9,04	4.73	104.80	13,46	5.60	96.12	14,27	2.98
78	Cyprodinil	0.20	0.9945	N/A	N/A	N/A	109.65	9,34	10.35	95.04	10,01	19.09	103.39	14,24	5.60	95.75	12,78	3.91
79	Cyromazine	2.00	0.9904	N/A	N/A	N/A	N/A	N/A	N/A	N/A	N/A	N/A	97.88	15,22	0.44	101.73	10,16	2.26
80	Danofloxacin	1.20	0.9698	N/A	N/A	N/A	N/A	N/A	N/A	87.87	15,46	4.73	88.06	9,23	1.32	95.42	16,22	21.72
81	Dazomet	1.60	0.9954	N/A	N/A	N/A	N/A	N/A	N/A	N/A	N/A	N/A	107.37	10,51	9.72	100.18	9,74	4.16
82	Deltamethrin	0.80	0.9927	N/A	N/A	N/A	N/A	N/A	N/A	104.72	7,30	11.36	118.20	12,06	2.60	98.42	8,92	1.75
83	Demeton-S-methyl	0.10	0.9966	113.64	12,45	4.39	98.56	13,75	7.34	91.34	8,63	1.66	100.20	14,19	7.48	95.83	12,90	0.52
84	Demeton-S-methyl-sulfone (Dioxydemeton)	0.40	0.9962	N/A	N/A	N/A	116.45	10,04	12.58	98.84	15,84	1.65	101.43	12,71	5.48	97.04	9,37	0.23
85	Dexamethasone	0.40	0.9920	N/A	N/A	N/A	129.03	9,03	9.22	100.41	12,98	4.52	102.29	13,81	11.14	94.51	10,33	0.39
86	Diazinon	0.40	0.9923	N/A	N/A	N/A	106.32	10,31	1.65	110.27	15,60	13.64	90.53	9,96	8.70	97.56	10,05	3.30
87	Dibenzo[a.h]anthracene	0.40	0.9884	N/A	N/A	N/A	122.87	13,55	14.78	106.79	7,27	6.03	95.70	13,68	10.20	100.26	14,21	9.02
88	Dichlorodiphenyldichloroethane (p.p’ DDD)	0.10	0.9783	93.23	6,40	7.21	95.01	10,77	9.24	115.50	12,04	6.52	88.01	14,59	9.38	86.76	10,58	7.80
89	Dichlorodiphenyldichloroethylene (p.p’ DDE)	0.10	0.9848	94.34	8,74	5.45	95.01	15,21	7.23	115.50	7,37	6.52	88.01	13,38	9.38	86.76	15,66	7.80
90	Dichlorodiphenyltrichloroethane (p.p’ DDT)	1.20	0.9740	N/A	N/A	N/A	N/A	N/A	N/A	124.05	7,26	4.15	82.09	11,36	19.34	97.14	12,26	17.84
91	Diclofenac	0.80	0.9677	N/A	N/A	N/A	N/A	N/A	N/A	79.69	9,75	18.79	104.25	15,50	6.86	104.76	14,33	0.55
92	Dicloran	0.10	0.9836	123.22	9,55	8.22	108.31	9,22	11.23	114.09	14,60	6.44	107.57	9,01	11.46	99.19	9,49	8.92
93	Dicloxacillin	1.20	0.9832	N/A	N/A	N/A	N/A	N/A	N/A	118.47	13,71	25.92	107.63	9,22	2.05	94.46	11,45	8.19
94	Dieldrin	1.20	0.9916	N/A	N/A	N/A	N/A	N/A	N/A	90.79	16,01	2.75	93.04	10,08	18.65	102.23	10,10	6.68
95	Diethathyl ethyl	0.20	0.9949	N/A	N/A	N/A	101.03	13,95	15.93	97.12	8,14	6.24	109.86	9,80	1.29	97.92	13,74	5.33
96	Diethofencarb	0.10	0.9974	109.28	9,41	9.22	99.69	10,24	14.33	90.50	16,03	5.04	103.94	14,12	11.39	97.29	9,31	3.12
97	Difenacoum	0.20	0.9821	N/A	N/A	N/A	106.25	12,62	0.67	97.13	7,96	1.76	89.59	15,15	10.58	101.79	12,53	13.24
98	Difenoconazole	0.40	0.9962	N/A	N/A	N/A	110.55	14,29	2.18	97.28	7,66	6.95	103.07	15,99	5.52	98.83	15,16	3.65
99	Difethialone	0.80	0.9710	N/A	N/A	N/A	N/A	N/A	N/A	95.73	7,39	17.88	91.34	16,61	15.31	102.52	12,84	22.89
100	Difloxacin	0.80	0.9702	N/A	N/A	N/A	N/A	N/A	N/A	74.73	16,49	11.56	95.04	9,89	3.47	97.27	10,00	24.41
101	Diflubenzuron	1.20	0.9909	N/A	N/A	N/A	N/A	N/A	N/A	82.74	13,96	13.97	104.38	14,08	2.84	97.93	9,13	8.15
102	Diflufenican	0.10	0.9943	104.94	10,18	11.58	95.70	10,05	10.45	85.39	14,12	4.30	110.99	9,10	5.78	99.81	9,32	0.67
103	Dimethenamid-P (and its R-isomer)	0.10	0.9963	127.37	6,66	4.97	101.55	13,43	3.07	91.45	8,20	11.35	91.32	9,36	16.95	96.98	14,60	0.53
104	Dimethoate	0.40	0.9967	N/A	N/A	N/A	122.10	9,22	0.83	98.13	15,13	4.07	102.92	10,29	6.04	97.11	11,18	0.20
105	Dimethomorph (two isomers)	0.40	0.9966	N/A	N/A	N/A	117.73	13,92	4.31	97.14	8,28	2.29	101.37	9,10	8.66	97.41	15,53	2.53
106	Dimethylphenylsulfamide (DMSA. metabolite of dichlofluanid)	0.80	0.9971	N/A	N/A	N/A	N/A	N/A	N/A	99.37	8,54	5.30	97.54	13,42	8.62	98.06	12,18	1.27
107	Diniconazole-M	0.20	0.9972	N/A	N/A	N/A	110.11	12,82	18.86	86.32	9,11	8.30	103.99	14,19	10.86	98.08	14,05	0.98
108	Dinocap	0.80	0.9847	N/A	N/A	N/A	**N/A**	N/A	**N/A**	90.93	15,41	5.14	102.73	15,20	10.95	95.70	9,66	8.61
109	Diphacinone	1.20	0.9892	N/A	N/A	N/A	N/A	N/A	N/A	115.88	13,56	4.43	105.36	16,11	8.05	98.59	9,73	5.59
110	Diphenylamine	0.20	0.9938	N/A	N/A	N/A	104.56	10,04	10.33	100.70	16,33	1.21	81.45	9,71	11.19	103.06	9,82	2.82
111	Dodine	0.40	0.9953	N/A	N/A	N/A	98.40	15,62	11.83	102.50	8,23	1.46	101.49	12,47	2.92	95.74	15,22	2.74
112	Endosulfan alfa	0.80	0.9931	N/A	N/A	N/A	N/A	N/A	N/A	101.76	12,52	13.79	86.34	9,89	13.22	100.60	9,22	2.10
113	Endosulfan beta	0.80	0.9859	N/A	N/A	N/A	N/A	N/A	N/A	116.18	8,01	17.11	94.87	8,97	15.61	102.32	12,87	8.66
114	Endosulfan sulfate	0.80	0.9884	N/A	N/A	N/A	N/A	N/A	N/A	111.36	7,97	4.46	99.14	10,28	12.76	101.10	13,93	4.21
115	Endrin	1.60	0.9961	N/A	N/A	N/A	N/A	N/A	N/A	N/A	N/A	N/A	105.62	8,92	11.25	83.94	14,41	7.55
116	Enrofloxacin	1.20	0.9569	N/A	N/A	N/A	N/A	N/A	N/A	90.24	14,45	1.61	79.66	13,76	16.02	92.99	11,50	34.07
117	EPN	0.80	0.9873	N/A	N/A	N/A	N/A	N/A	N/A	100.07	16,63	3.78	94.94	15,70	17.91	98.99	9,19	2.59
118	Epoxiconazole	0.20	0.9966	N/A	N/A	N/A	109.95	10,50	2.71	87.76	17,90	15.32	99.14	13,04	5.31	97.64	9,13	1.60
119	Eprinomectin	0.20	0.9878	N/A	N/A	N/A	121.34	13,63	9.72	99.91	7,56	12.53	112.05	8,92	1.98	106.09	11,98	0.52
120	Eritromicin	0.20	0.9967	N/A	N/A	N/A	105.88	10,86	6.29	96.88	16,44	3.65	103.97	9,77	6.11	96.79	10,70	3.68
121	Esfenvalerate	2.00	0.9936	N/A	N/A	N/A	N/A	N/A	N/A	N/A	N/A	N/A	96.38	10,37	3.14	96.89	13,24	5.12
122	Ethion (diethion)	0.10	0.9958	103.66	7,72	0.78	89.88	15,17	3.73	95.28	7,88	3.77	108.49	8,87	4.58	100.49	15,28	4.16
123	Ethirimol	0.40	0.9964	N/A	N/A	N/A	122.23	12,67	1.90	96.57	12,01	0.27	104.84	12,91	3.65	98.37	13,21	4.56
124	Ethofumesate	0.80	0.9846	N/A	N/A	N/A	N/A	N/A	N/A	91.55	10,20	4.97	82.64	18,27	14.03	98.52	10,25	11.95
125	Ethoprophos	0.20	0.9953	N/A	N/A	N/A	96.56	9,60	16.85	96.29	10,50	4.92	102.00	14,06	7.17	95.03	11,29	2.62
126	Etofenprox	0.80	0.9963	N/A	N/A	N/A	N/A	N/A	N/A	98.75	10,23	4.30	93.90	15,00	7.98	100.65	9,10	5.37
127	Etoxazole	0.10	0.9972	106.66	6,86	18.17	87.77	16,23	9.12	91.35	9,95	7.34	103.72	9,01	3.18	100.75	13,94	4.65
128	Famoxadone	1.20	0.9942	N/A	N/A	N/A	N/A	N/A	N/A	109.69	9,40	14.42	101.73	12,93	9.40	98.87	10,38	3.93
129	Fenamidone	0.10	0.9976	100.84	9,44	2.17	102.38	12,60	9.41	99.89	9,41	9.34	105.57	9,85	4.55	97.86	13,99	2.26
130	Fenamiphos	0.10	0.9976	92.87	15,36	1.25	94.57	14,70	9.17	99.28	8,99	1.65	107.18	9,85	5.85	95.32	13,62	3.93
131	Fenamiphos sulfone	0.20	0.9971	N/A	N/A	N/A	109.57	13,30	6.60	90.42	10,49	0.60	103.95	8,98	7.13	96.59	15,51	1.85
132	Fenamiphos sulfoxide	0.40	0.9963	N/A	N/A	N/A	120.76	9,74	4.40	96.93	9,25	3.43	99.10	9,42	7.16	97.96	10,91	0.25
133	Fenarimol	0.20	0.9960	N/A	N/A	N/A	107.08	10,55	8.23	100.38	7,97	1.42	91.15	14,84	3.62	98.31	9,95	0.95
134	Fenazaquin	0.80	0.9967	N/A	N/A	N/A	N/A	N/A	N/A	103.00	7,70	4.49	107.95	15,93	4.82	99.17	17,83	1.81
135	Fenbendazole	0.10	0.9959	117.39	10,22	0.85	96.83	15,21	7.43	92.86	10,50	12.54	111.90	15,15	3.55	96.47	6,84	0.78
136	Fenbuconazole	0.40	0.9943	N/A	N/A	N/A	106.97	10,09	11.96	97.78	8,02	4.11	82.91	16,31	2.82	96.67	17,54	4.23
137	Fenbutatin oxide	0.80	0.9705	N/A	N/A	N/A	N/A	N/A	N/A	101.12	10,91	2.09	101.73	10,08	2.23	109.08	6,62	3.83
138	Fenhexamid	1.60	0.9952	N/A	N/A	N/A	N/A	N/A	N/A	N/A	N/A	N/A	94.50	12,93	5.93	100.97	6,91	1.63
139	Fenitrothion	0.20	0.9946	N/A	N/A	N/A	96.00	13,47	1.84	104.50	10,63	18.04	89.65	10,40	10.92	98.77	6,63	0.21
140	Fenoxycarb	0.10	0.9950	106.29	15,35	14.48	89.97	9,71	10.85	88.98	8,95	0.61	108.29	14,13	1.92	101.10	17,34	1.20
141	Fenpropathrin	0.40	0.9932	N/A	N/A	N/A	96.77	9,48	4.11	108.60	7,36	7.89	114.09	15,57	4.22	98.64	14,32	2.46
142	Fenpropidin	0.10	0.9971	107.57	7,02	6.44	95.01	9,25	14.96	95.57	8,20	6.27	104.76	14,60	3.69	98.78	16,42	2.87
143	Fenpropimorph	0.10	0.9967	112.10	6,69	5.06	91.37	15,36	6.27	98.02	11,73	3.30	105.01	7,29	4.62	98.31	7,08	2.04
144	Fenpyroximate	0.40	0.9972	N/A	N/A	N/A	108.10	9,24	11.74	98.03	9,60	3.42	110.63	7,30	7.96	99.89	14,81	3.51
145	Fenthion	0.10	0.9907	101.39	8,20	9.98	98.48	14,20	6.59	100.84	10,07	1.09	93.69	7,24	3.06	97.74	6,90	2.60
146	Fenthion oxon	0.10	0.9981	102.81	9,49	11.00	93.65	12,81	5.77	95.62	9,66	2.50	103.73	7,29	4.97	97.83	6,63	1.80
147	Fenthion oxon sulfone	0.80	0.9978	N/A	N/A	N/A	N/A	N/A	N/A	99.68	9,78	2.02	101.55	16,22	6.88	98.19	6,93	2.88
148	Fenthion oxon sulfoxide	0.20	0.9977	N/A	N/A	N/A	119.70	9,46	1.24	99.13	9,09	5.16	101.41	7,40	5.89	97.91	18,80	0.84
149	Fenthion sulfone	0.80	0.9980	N/A	N/A	N/A	N/A	N/A	N/A	102.66	8,33	0.50	101.78	13,19	9.01	98.32	17,66	2.07
150	Fenthion sulfoxide	0.40	0.9991	N/A	N/A	N/A	106.77	10,32	6.95	103.30	9,24	8.77	103.57	16,11	4.88	95.47	15,28	1.90
151	Fenvalerate	2.00	0.9939	N/A	N/A	N/A	N/A	N/A	N/A	N/A	N/A	N/A	91.05	15,63	10.82	98.09	6,62	1.48
152	Fipronil	0.20	0.9946	N/A	N/A	N/A	104.05	9,16	2.31	90.35	10,22	2.29	101.81	13,15	4.10	100.66	15,46	0.15
153	Fipronil sulfide	0.80	0.9391	N/A	N/A	N/A	N/A	N/A	N/A	98.90	11,00	8.97	84.35	7,92	13.73	95.07	7,23	27.31
154	Flocoumafen	0.20	0.9737	N/A	N/A	N/A	115.03	13,55	2.97	97.42	10,83	10.78	81.77	8,58	6.71	99.46	6,62	19.13
155	Fluazinam	0.20	0.9942	N/A	N/A	N/A	109.04	13,34	9.01	95.48	10,76	4.20	101.92	7,31	1.50	96.92	6,62	2.29
156	Flubendiamide	2.00	0.9829	N/A	N/A	N/A	N/A	N/A	N/A	N/A	N/A	N/A	91.98	8,33	10.12	95.45	17,55	9.10
157	Flucythrinate (two isomers)	0.80	0.9951	N/A	N/A	N/A	N/A	N/A	N/A	100.00	9,93	2.37	93.42	16,52	4.71	99.66	14,19	4.20
158	Fludioxonil	0.20	0.9924	N/A	N/A	N/A	99.12	9,56	6.85	96.89	9,31	6.19	88.02	7,25	3.14	98.96	17,89	9.67
159	Flufenoxuron	0.10	0.9942	107.97	6,05	14.68	88.19	14,34	10.25	88.31	12,00	4.19	112.03	7,62	4.34	102.01	6,75	2.92
160	Flumequine	0.10	0.9876	106.13	8,91	23.04	100.69	11,01	10.95	91.44	8,68	9.51	96.97	9,32	10.04	91.89	15,61	17.35
161	Flunixin	0.20	0.9949	N/A	N/A	N/A	96.90	14,66	4.63	91.59	9,37	1.62	110.14	7,43	2.78	98.17	6,63	0.65
162	Fluopyram	0.20	0.9958	N/A	N/A	N/A	110.51	12,65	3.31	97.01	11,93	0.48	94.66	9,95	8.92	96.17	6,77	6.46
163	Fluoranthene	0.20	0.9887	N/A	N/A	N/A	109.74	13,17	6.76	104.20	11,02	5.89	94.00	7,25	10.02	85.54	7,48	7.69
164	Fluorene	0.20	0.9836	N/A	N/A	N/A	148.49	9,00	13.22	111.31	8,80	6.29	92.49	16,86	9.86	93.39	17,74	8.40
165	Fluquinconazole	0.20	0.9869	N/A	N/A	N/A	103.74	10,15	12.58	96.88	8,10	4.65	88.44	17,22	1.18	96.68	17,03	13.29
166	Flusilazole	0.20	0.9946	N/A	N/A	N/A	94.54	9,43	10.00	89.82	10,64	0.48	114.38	15,47	2.02	98.57	14,14	2.32
167	Flutolanil	0.10	0.9975	106.84	7,89	11.91	89.48	13,65	6.56	100.18	11,64	5.67	106.33	16,01	3.74	98.51	6,62	2.19
168	Flutriafol	0.20	0.9953	N/A	N/A	N/A	96.42	10,14	7.37	105.15	9,45	8.63	90.02	8,07	2.90	96.88	17,53	0.89
169	Fluvalinate tau	4.00	0.9356	N/A	N/A	N/A	N/A	N/A	N/A	N/A	N/A	N/A	90.97	7,25	5.31	106.96	6,69	10.74
170	Fonofos	0.40	0.9924	N/A	N/A	N/A	104.18	13,29	14.80	101.25	11,79	6.87	87.63	7,29	10.09	97.22	6,69	4.61
171	Formetanate	0.10	0.9967	106.33	13,02	9.84	96.75	14,29	0.48	94.60	10,34	0.85	109.06	7,56	4.34	100.01	6,95	5.73
172	Fosthiazate	0.10	0.9972	106.58	11,43	10.48	97.01	9,22	10.28	95.35	8,80	6.45	101.87	15,78	5.69	96.64	14,66	1.77
173	Heptachlor	0.80	0.9722	N/A	N/A	N/A	N/A	N/A	N/A	87.12	8,39	4.92	139.37	7,28	14.85	92.22	14,83	8.29
174	Hexachlorobencene	0.20	0.9858	N/A	N/A	N/A	99.79	9,37	9.45	105.21	8,36	5.94	93.97	16,62	10.01	93.24	15,11	8.38
175	Hexachlorocyclohexane (alpha)	0.40	0.9818	N/A	N/A	N/A	N/A	N/A	N/A	104.74	10,64	5.92	104.70	13,30	11.16	102.65	6,65	9.23
176	Hexachlorocyclohexane (beta)	0.40	0.9456	N/A	N/A	N/A	N/A	N/A	N/A	113.08	7,52	6.39	132.60	13,78	14.13	109.11	14,55	9.81
177	Hexachlorocyclohexane (delta)	0.20	0.9803	N/A	N/A	N/A	78.98	14,62	7.89	103.75	10,97	5.86	108.30	13,54	11.54	100.42	6,69	9.03
178	Hexaclorocyclohexane (gamma. lindane)	1.20	0.9788	N/A	N/A	N/A	N/A	N/A	N/A	74.44	10,23	4.20	116.82	7,46	12.45	109.76	6,66	9.87
179	Hexaconazole (two isomers)	0.80	0.9956	N/A	N/A	N/A	N/A	N/A	N/A	102.27	13,88	4.57	99.87	7,28	3.77	100.02	6,65	4.07
180	Hexaflumuron	0.40	0.9942	N/A	N/A	N/A	108.89	9,42	12.82	90.01	9,26	4.34	105.96	7,25	4.78	101.95	16,19	1.84
181	Hexythiazox	0.10	0.9901	100.56	8,48	12.26	92.79	9,89	0.73	88.30	9,01	12.34	108.98	7,31	4.58	101.78	15,67	2.78
182	Imazalil (enilconazole)	0.40	0.9962	N/A	N/A	N/A	109.11	10,51	1.10	99.07	9,60	0.11	105.22	14,79	1.79	100.07	17,30	5.52
183	Imidacloprid	0.80	0.9952	N/A	N/A	N/A	N/A	N/A	N/A	87.81	15,08	2.99	104.63	7,28	11.64	96.04	6,63	2.58
184	Indeno [1.2.3-cd] pyrene	0.40	0.9919	N/A	N/A	N/A	N/A	N/A	N/A	104.74	9,05	5.92	104.70	13,91	11.16	102.65	15,55	9.23
185	Indoxacarb	0.20	0.9936	N/A	N/A	N/A	107.04	14,47	9.81	90.81	17,33	0.63	104.94	16,20	0.90	100.08	6,69	2.82
186	Iprodione	4.00	0.9910	N/A	N/A	N/A	N/A	N/A	N/A	N/A	N/A	N/A	92.28	16,39	18.00	99.85	6,62	5.16
187	Iprovalicarb	0.20	0.9976	N/A	N/A	N/A	104.86	13,80	1.64	96.91	14,24	4.68	104.31	6,64	5.23	99.02	7,01	2.18
188	Isocarbophos	1.60	0.9921	N/A	N/A	N/A	N/A	N/A	N/A	N/A	N/A	N/A	97.94	14,54	9.86	101.76	14,94	7.87
189	Isofenphos methyl	0.40	0.9915	N/A	N/A	N/A	109.93	9,31	13.09	103.10	9,03	4.81	90.82	16,19	8.17	97.95	17,33	9.64
190	Isoprothiolane	0.10	0.9964	116.56	8,05	1.22	88.49	11,32	14.49	97.12	9,55	1.20	104.61	17,73	4.03	98.25	16,45	3.16
191	Ivermectin B1a	1.60	0.9537	N/A	N/A	N/A	N/A	N/A	N/A	N/A	N/A	N/A	105.65	17,48	10.35	105.44	6,85	17.66
192	Josamycin	0.40	0.9963	N/A	N/A	N/A	116.58	9,84	9.00	103.29	9,03	8.32	100.89	6,75	3.99	97.02	14,41	4.09
193	Ketoprofen	0.40	0.9947	N/A	N/A	N/A	111.09	13,69	9.48	91.37	15,03	5.33	104.97	16,90	2.64	95.12	6,88	4.68
194	Kresoxim methyl	1.20	0.9954	N/A	N/A	N/A	N/A	N/A	N/A	102.98	15,93	5.45	88.44	6,70	7.22	96.75	6,67	3.56
195	Leptophos	0.80	0.9941	N/A	N/A	N/A	N/A	N/A	N/A	101.99	16,06	1.71	98.45	6,98	9.13	98.20	6,83	6.65
196	Levamisole	0.20	0.9928	N/A	N/A	N/A	117.03	9,11	15.55	93.61	9,12	5.55	101.49	6,99	1.47	94.01	15,02	3.40
197	Lincomycin	0.40	0.9954	N/A	N/A	N/A	107.94	10,04	20.27	92.20	9,04	10.96	106.37	6,74	7.35	97.78	16,09	7.36
198	Linuron	0.20	0.9950	N/A	N/A	N/A	111.70	9,18	8.45	96.83	9,31	11.03	104.59	17,99	2.83	97.46	13,85	1.94
199	Lufenuron	0.40	0.9451	N/A	N/A	N/A	124.88	12,48	1.73	104.02	14,96	4.07	76.66	19,41	9.50	108.83	6,75	6.81
200	Malaoxon	0.10	0.9968	105.72	13,29	7.33	98.47	10,73	10.38	93.12	9,21	5.62	103.41	6,91	5.54	96.61	13,88	1.87
201	Malathion	0.20	0.9956	N/A	N/A	N/A	102.68	15,17	2.06	108.09	15,52	6.64	108.81	6,62	6.47	95.13	6,74	1.66
202	Mandipropamid	0.10	0.9965	115.12	12,98	1.19	88.72	12,74	4.12	94.24	16,17	0.83	104.13	6,65	1.48	97.38	6,62	1.67
203	Marbofloxacin	2.00	0.9589	N/A	N/A	N/A	N/A	N/A	N/A	N/A	N/A	N/A	58.20	8,81	13.63	95.33	6,67	21.85
204	Mebendazole	0.10	0.9960	139.72	14,34	0.62	93.53	9,81	2.13	93.17	9,63	5.68	104.77	16,51	3.77	97.25	14,46	2.70
205	Mefenamic acid	0.40	0.9938	N/A	N/A	N/A	111.93	10,36	4.50	97.29	9,33	11.22	106.39	15,98	0.91	101.74	16,63	0.41
206	Mefenoxam (metalaxyl-M)	0.10	0.9974	104.72	14,28	6.08	101.99	9,15	10.56	95.87	9,02	4.94	103.12	15,00	7.93	96.94	15,42	2.84
207	Meloxicam	0.20	0.9916	N/A	N/A	N/A	105.76	13,99	8.34	103.10	16,06	8.66	103.87	15,74	0.35	95.21	6,62	7.62
208	Mepanipyrim	0.40	0.9940	N/A	N/A	N/A	112.41	9,22	16.49	100.23	9,21	5.60	86.88	7,31	4.08	98.52	18,16	5.19
209	Mepiquat	0.40	0.9961	N/A	N/A	N/A	116.63	13,85	16.83	102.27	15,30	6.19	109.64	15,10	6.19	98.75	6,68	1.89
210	Metaflumizone	0.20	0.9538	N/A	N/A	N/A	117.95	13,04	7.80	110.07	14,54	14.54	78.67	7,94	1.92	108.66	7,10	15.31
211	Metaldehyde	4.00	0.9883	N/A	N/A	N/A	N/A	N/A	N/A	N/A	N/A	N/A	93.85	6,69	11.49	99.36	7,06	5.12
212	Metconazole	0.10	0.9972	112.89	17,30	7.83	90.40	10,89	3.31	94.39	9,51	3.75	106.51	6,73	5.49	99.30	7,31	1.11
213	Methamidophos (two isomers)	1.20	0.9914	N/A	N/A	N/A	N/A	N/A	N/A	106.70	9,02	11.46	118.60	6,73	4.89	100.44	7,26	3.27
214	Methidathion	0.10	0.9971	103.57	9,74	14.60	93.56	11,22	7.71	97.65	9,08	0.47	105.01	15,31	4.15	98.65	7,31	3.48
215	Methiocarb	0.10	0.9988	102.06	8,49	8.48	80.51	15,96	14.62	104.48	15,11	0.95	102.47	15,17	6.24	99.13	15,56	0.40
216	Methiocarb-sulfoxide	0.80	0.9983	N/A	N/A	N/A	N/A	N/A	N/A	106.81	9,41	3.29	100.84	16,38	3.36	99.34	7,92	2.12
217	Methomyl	0.40	0.9957	N/A	N/A	N/A	123.44	12,82	2.09	97.84	14,07	5.96	103.37	18,15	3.88	97.62	16,66	3.36
218	Methomyl oxime	8.00	0.9878	N/A	N/A	N/A	N/A	N/A	N/A	N/A	N/A	N/A	N/A	N/A	N/A	104.43	16,22	11.23
219	Methoxyfenozide	0.10	0.9970	101.72	8,47	12.57	94.61	12,59	7.91	92.06	16,58	4.97	107.21	16,10	4.63	98.53	14,79	5.72
220	Metoxychlor	0.80	0.9654	N/A	N/A	N/A	N/A	N/A	N/A	103.00	9,01	4.49	107.95	6,76	4.82	99.17	7,25	1.81
221	Metrafenone	0.10	0.9961	105.81	19,69	4.81	96.19	10,87	16.34	92.34	9,58	1.92	104.28	6,62	2.67	97.90	7,25	4.32
222	Metronidazole	0.80	0.9962	N/A	N/A	N/A	N/A	N/A	N/A	98.63	9,76	3.50	102.00	6,68	8.71	95.00	7,37	3.36
223	Mevinphos (phosdrin)	0.80	0.9915	N/A	N/A	N/A	N/A	N/A	N/A	107.53	13,72	1.76	101.38	6,92	3.71	96.27	16,29	4.50
224	Mirex	2.00	0.9549	N/A	N/A	N/A	N/A	N/A	N/A	N/A	N/A	N/A	94.53	15,92	10.07	80.16	9,18	7.21
225	Monocrotophos	0.80	0.9965	N/A	N/A	N/A	N/A	N/A	N/A	103.97	8,42	2.26	101.05	18,14	3.31	98.38	16,59	0.70
226	Myclobutanil	0.10	0.9951	124.02	8,55	14.29	90.40	14,35	1.18	93.06	10,29	11.51	104.50	14,50	1.48	97.54	14,00	1.84
227	N-(2.4-dimethylphenyl)-N’-methylformamidine (DMPF. metabolite of amitraz)	0.80	0.9974	N/A	N/A	N/A	N/A	N/A	N/A	97.86	8,07	10.59	103.78	14,78	4.26	97.74	15,81	2.58
228	N.N-dimethylformamidine (DMF. metabolite of amitraz)	1.20	0.9835	N/A	N/A	N/A	N/A	N/A	N/A	100.61	13,54	3.89	89.22	6,62	8.12	94.96	7,43	5.62
229	N.N-Dimethyl-N'-p-tolylsulphamide (DMST. metabolite of tolyfluanid)	0.20	0.9972	N/A	N/A	N/A	103.04	10,88	5.68	93.42	10,84	1.84	103.03	16,30	5.97	96.86	7,99	1.30
230	Nafcillin	0.80	0.9937	N/A	N/A	N/A	N/A	N/A	N/A	88.13	14,49	4.44	108.84	6,82	0.54	103.15	7,68	2.36
231	Naphtalene	0.80	0.9321	N/A	N/A	N/A	N/A	N/A	N/A	101.12	8,54	2.09	101.73	6,83	2.23	109.08	14,76	3.83
232	Naproxen	1.60	0.9915	N/A	N/A	N/A	N/A	N/A	N/A	N/A	N/A	N/A	103.39	6,66	13.15	101.78	7,24	7.98
233	Nitenpyram	2.00	0.9983	N/A	N/A	N/A	N/A	N/A	N/A	N/A	N/A	N/A	95.74	12,88	1.32	98.39	13,54	2.80
234	Novobiocin	0.80	0.9906	N/A	N/A	N/A	N/A	N/A	N/A	103.08	7,97	18.91	105.94	9,44	19.94	94.20	16,68	2.89
235	Nuarimol	0.20	0.9907	N/A	N/A	N/A	109.73	12,42	0.51	97.78	10,16	3.72	90.15	10,54	2.92	99.33	14,45	13.10
236	Ofurace	0.10	0.9950	116.80	13,54	2.28	101.16	9,23	4.08	92.59	10,84	1.49	103.20	9,09	6.63	96.53	7,41	0.65
237	Omethoate	0.40	0.9955	N/A	N/A	N/A	123.46	9,26	4.50	92.85	14,57	13.56	103.56	14,05	4.38	97.89	7,34	1.03
238	Oxadixyl	0.20	0.9968	N/A	N/A	N/A	104.76	9,57	3.73	95.70	11,92	0.86	103.01	13,24	5.95	95.73	7,27	2.17
239	Oxamyl	0.40	0.9963	N/A	N/A	N/A	121.94	12,38	6.94	100.43	9,24	6.59	100.86	15,07	1.70	97.44	13,92	1.86
240	Oxfendazole	0.10	0.9958	127.49	9,14	3.24	103.94	9,83	7.55	89.18	10,70	3.62	100.67	15,04	7.94	96.27	7,42	1.82
241	Oxolinic acid	0.20	0.9878	N/A	N/A	N/A	115.69	14,38	16.83	83.19	8,62	3.27	93.60	11,38	7.48	91.97	13,85	12.34
242	Oxydemeton methyl	0.40	0.9957	N/A	N/A	N/A	121.11	14,05	6.52	92.99	8,25	1.58	101.52	9,52	5.54	97.61	15,88	2.85
243	Oxyfluorfen	0.40	0.9951	N/A	N/A	N/A	111.90	12,27	10.58	104.83	9,18	6.06	89.81	11,13	8.87	97.95	15,20	6.39
244	Paclobutrazol	0.40	0.9967	N/A	N/A	N/A	116.18	9,54	1.19	93.47	14,62	0.60	101.83	9,58	2.63	98.88	7,40	0.81
245	Paraoxon methyl	1.60	0.9967	N/A	N/A	N/A	N/A	N/A	N/A	N/A	N/A	N/A	87.46	14,41	19.55	101.53	7,25	6.01
246	Parathion ethyl	1.20	0.9566	N/A	N/A	N/A	N/A	N/A	N/A	104.49	13,49	4.82	89.57	9,37	14.20	96.19	7,67	3.90
247	Parathion methyl	0.80	0.9976	N/A	N/A	N/A	N/A	N/A	N/A	108.54	9,72	8.23	92.53	12,51	19.31	100.15	13,94	12.02
248	PCB 28	0.10	0.9912	83.22	13,04	15.78	93.92	10,39	11.89	106.19	11,02	6.00	97.60	12,20	10.40	88.62	7,25	7.97
249	PCB 52	0.20	0.9902	N/A	N/A	N/A	104.04	15,11	10.65	98.92	8,87	5.59	92.35	15,12	9.84	93.96	13,69	8.45
250	PCB 77	0.20	0.9923	N/A	N/A	N/A	100.53	14,86	8.65	98.89	9,22	5.59	88.02	16,35	9.38	92.40	14,65	8.31
251	PCB 81	0.10	0.9850	110.87	13,67	12.33	81.87	13,56	7.45	115.62	7,80	6.53	91.34	9,29	9.73	89.92	14,75	8.09
252	PCB 101	0.20	0.9876	N/A	N/A	N/A	107.84	10,20	12.34	110.75	10,27	6.26	93.40	10,87	9.95	92.89	7,29	8.35
253	PCB 105	0.10	0.9789	118.45	12,59	4.12	103.80	9,79	9.54	107.96	10,72	6.10	94.53	9,54	10.07	80.16	7,58	7.21
254	PCB 114	0.20	0.9776	N/A	N/A	N/A	97.14	10,43	6.22	110.95	11,13	6.27	85.53	11,13	9.11	84.13	8,29	7.57
255	PCB 118	0.20	0.9833	N/A	N/A	N/A	93.43	15,84	9.87	116.88	9,41	6.60	88.80	14,30	9.46	86.10	16,14	7.74
256	PCB 123	0.40	0.9834	N/A	N/A	N/A	96.14	9,55	11.67	106.30	12,46	6.01	88.58	9,79	9.44	85.35	7,25	7.67
257	PCB 126	0.20	0.9789	N/A	N/A	N/A	108.37	12,34	12.09	106.86	8,54	6.04	90.82	15,17	9.68	79.34	11,78	7.13
258	PCB 138	0.10	0.9798	107.23	9,04	9.56	91.30	13,54	9.07	110.40	8,31	6.24	92.83	14,93	9.89	84.08	9,09	7.56
259	PCB 153	0.10	0.9766	117.78	9,83	8.73	83.70	15,17	4.97	124.77	9,02	7.05	89.77	15,53	9.57	85.65	10,85	7.70
260	PCB 156	0.20	0.9912	N/A	N/A	N/A	98.33	10,47	7.21	107.17	16,36	6.05	91.74	15,28	9.78	77.11	17,71	6.93
261	PCB 157	0.40	0.9789	N/A	N/A	N/A	93.22	10,60	6.54	107.96	15,32	6.10	91.26	9,70	9.72	79.99	15,86	7.19
262	PCB 167	0.10	0.9770	102.89	9,26	6.45	91.43	11,68	8.97	102.06	14,16	5.77	86.31	9,26	9.20	81.14	15,29	7.30
263	PCB 169	0.20	0.9758	N/A	N/A	N/A	109.07	12,62	11.56	107.45	9,51	6.07	86.95	10,36	9.26	90.77	9,14	8.16
264	PCB 180	0.10	0.9807	119.51	8,34	20.01	87.43	12,13	10.76	113.14	15,94	6.39	85.21	11,75	9.08	95.03	13,54	8.55
265	PCB 189	0.10	0.9723	110.12	8,68	12.45	97.91	15,11	6.22	96.96	9,32	5.48	88.62	12,49	9.44	87.18	9,24	7.84
266	Penconazole	0.40	0.9930		N/A		114.19	13,25	14.47	101.18	9,00	3.31	91.43	10,09	1.78	99.02	9,49	10.75
267	Pencycuron	0.10	0.9968	97.11	8,84	9.23	94.99	12,74	13.51	93.82	9,10	3.87	105.44	12,04	6.20	97.74	9,16	0.70
268	Pendimethalin	0.80	0.9902	N/A	N/A	N/A	N/A	N/A	N/A	106.58	16,84	2.61	88.47	15,67	3.62	97.69	16,40	0.06
269	Penicillin G	2.00	0.9903	N/A	N/A	N/A	N/A	N/A	N/A	N/A	N/A	N/A	117.02	13,91	3.71	97.77	12,92	3.62
270	Penicillin V	2.00	0.9917	N/A	N/A	N/A	N/A	N/A	N/A	N/A	N/A	N/A	105.81	13,39	2.98	97.69	15,53	3.59
271	Permethrin	1.20	0.9889	N/A	N/A	N/A	N/A	N/A	N/A	101.46	9,10	0.12	87.27	10,84	2.23	101.87	9,16	17.84
272	Phenanthrene	0.20	0.9678	N/A	N/A	N/A	109.89	10,49	13.76	106.65	16,96	6.02	92.56	10,39	9.86	95.32	14,63	8.57
273	Phenylbutazone	1.60	0.9721	N/A	N/A	N/A	N/A	N/A	N/A	N/A	N/A	N/A	113.13	9,97	6.52	65.14	12,76	6.05
274	Phosalone	0.20	0.9961	N/A	N/A	N/A	98.37	13,12	4.61	92.71	10,27	2.58	107.98	9,30	4.93	101.62	8,94	1.06
275	Phosmet	0.20	0.9972	N/A	N/A	N/A	98.92	13,74	3.15	93.00	9,82	2.84	104.52	14,39	5.06	98.67	9,58	1.56
276	Phosmet oxon	0.20	0.9993	N/A	N/A	N/A	95.79	10,14	7.39	102.23	14,85	5.34	102.16	9,29	5.78	99.08	15,44	1.95
277	Piperacillin	0.40	0.9793	N/A	N/A	N/A	N/A	N/A	N/A	N/A	N/A	N/A	96.34	11,70	1.45	98.60	15,17	6.10
278	Pirimicarb	0.10	0.9977	105.98	8,04	2.51	98.18	10,71	6.09	95.11	15,17	5.23	102.97	10,72	4.86	97.54	15,39	1.81
279	Pirimiphos ethyl	0.10	0.9883	104.58	15,84	12.37	98.69	14,95	9.94	97.93	9,00	0.38	104.83	10,74	4.05	98.96	9,62	2.64
280	Pirimiphos methyl	0.10	0.9944	112.73	15,00	0.58	93.49	10,21	1.96	106.42	14,32	3.74	89.01	9,11	3.18	98.28	16,06	5.62
281	Prochloraz	0.10	0.9947	125.26	18,00	18.57	91.55	13,09	1.08	87.91	9,80	7.63	100.57	9,08	13.10	97.13	9,11	2.15
282	Procymidone	1.60	0.9947	N/A	N/A	N/A	N/A	N/A	N/A	N/A	N/A	N/A	80.19	15,87	6.54	100.94	9,20	5.65
283	Profenofos	0.10	0.9941	111.06	7,48	4.52	92.80	15,13	3.54	96.49	9,29	1.38	106.87	14,90	3.35	98.58	9,03	3.48
284	Propamocarb	0.40	0.9959	N/A	N/A	N/A	122.72	9,79	9.24	97.58	15,13	4.21	97.03	14,92	10.35	96.69	14,18	1.39
285	Propargite	0.10	0.9936	100.21	8,65	2.94	95.89	9,50	5.23	96.17	15,13	7.55	109.05	11,86	5.19	100.09	15,81	2.82
286	Propiconazole	0.40	0.9932	N/A	N/A	N/A	130.04	9,40	1.49	99.77	14,15	3.82	102.03	10,74	0.34	99.32	14,78	0.40
287	Propoxur	0.10	0.9941	118.68	11,02	14.25	92.88	13,46	13.27	90.94	9,34	1.25	101.89	9,97	6.43	96.39	8,93	1.71
288	Propyzamide (pronamide)	0.10	0.9955	135.09	10,13	25.43	82.62	12,64	4.33	89.32	18,24	8.08	102.57	9,70	4.21	98.22	15,95	2.04
289	Proquinazid	0.20	0.9808	N/A	N/A	N/A	121.75	13,90	4.37	100.38	9,85	10.36	89.96	11,39	7.16	96.95	9,40	16.39
290	Prothioconazol	0.40	0.9935	N/A	N/A	N/A	103.16	14,09	9.30	100.90	9,00	8.76	87.83	15,40	1.26	96.35	10,44	0.81
291	Prothiophos	0.40	0.9912	N/A	N/A	N/A	179.86	11,21	33.27	107.75	9,12	5.60	91.21	10,22	8.06	97.75	10,50	11.76
292	Pymetrozine	0.80	0.9952	N/A	N/A	N/A	N/A	N/A	N/A	102.89	14,39	1.86	99.73	12,22	9.64	98.84	12,75	0.26
293	Pyraclostrobin	0.10	0.9970	105.05	8,46	0.95	88.71	11,51	20.65	95.80	15,56	0.93	104.40	13,96	8.09	97.82	13,51	0.60
294	Pyrazophos	0.10	0.9965	111.64	16,39	0.54	98.23	9,29	17.84	95.77	15,41	6.78	107.24	13,69	3.11	99.83	15,58	2.60
295	Pyrene	0.20	0.9880	N/A	N/A	N/A	96.42	13,97	9.56	93.61	9,19	5.29	93.37	13,91	9.95	91.07	9,54	8.19
296	Pyridaben	0.10	0.9973	105.99	9,65	29.54	86.82	10,63	8.41	97.27	11,31	14.36	111.71	10,40	5.14	100.32	13,91	1.83
297	Pyridaphenthion	0.20	0.9970	N/A	N/A	N/A	108.64	13,78	2.34	91.81	11,67	10.38	105.65	10,12	9.67	98.59	8,97	0.24
298	Pyrimethanil	0.20	0.9944	N/A	N/A	N/A	107.96	13,00	6.86	96.98	11,36	3.25	89.29	10,21	11.63	98.03	13,12	0.85
299	Pyriproxifen	0.10	0.9947	104.25	19,77	1.21	99.97	13,33	9.48	94.10	12,89	1.20	107.21	9,99	2.85	100.77	13,76	4.72
300	Quinalfos	0.20	0.9958	N/A	N/A	N/A	97.53	9,47	17.05	90.97	12,09	1.01	106.48	12,86	0.09	100.08	9,35	5.66
301	Quinoxyfen	0.10	0.9874	112.39	6,66	4.49	109.15	9,19	13.38	93.18	11,12	13.57	104.94	9,62	6.76	102.76	9,18	7.44
302	Rifampicin	0.80	0.9826	N/A	N/A	N/A	N/A	N/A	N/A	111.21	10,43	10.64	95.28	14,86	0.19	89.73	11,01	5.48
303	Rotenone	0.40	0.9929	N/A	N/A	N/A	117.98	13,24	31.54	89.20	12,85	5.35	113.57	12,47	3.51	100.90	15,11	3.15
304	Roxithromycin	0.80	0.9971	N/A	N/A	N/A	N/A	N/A	N/A	96.18	9,47	3.69	98.39	9,30	4.00	100.05	9,10	0.03
305	Sarafloxacin	4.00	0.9312	N/A	N/A	N/A	N/A	N/A	N/A	N/A	N/A	N/A	80.98	10,55	13.63	97.00	14,62	22.19
306	Simazine	0.20	0.9968	N/A	N/A	N/A	90.28	16,27	2.78	96.46	11,65	1.79	102.84	13,27	6.50	97.85	15,10	1.76
307	Spinosad (two isomers)	0.10	0.9970	119.58	11,44	9.10	84.56	15,73	4.96	94.98	13,13	9.89	100.78	16,05	7.72	96.81	12,98	1.95
308	Spiramycin (two isomers)	0.40	0.9915	N/A	N/A	N/A	131.47	9,05	5.94	95.33	9,51	14.39	106.27	13,21	4.78	97.03	10,02	3.05
309	Spirodiclofen	0.80	0.9869	N/A	N/A	N/A	N/A	N/A	N/A	106.47	10,78	1.93	134.35	14,32	8.81	100.48	10,43	7.70
310	Spiromesifen	0.20	0.9911	N/A	N/A	N/A	96.47	9,96	13.07	101.29	9,33	5.45	115.81	8,86	4.16	100.68	10,51	2.72
311	Spiroxamine	0.10	0.9972	107.65	10,26	2.69	97.02	13,19	5.23	95.80	13,52	6.32	102.89	14,63	3.99	97.41	13,84	4.04
312	Strychnine	0.80	0.9963	N/A	N/A	N/A	N/A	N/A	N/A	85.40	12,44	3.11	111.08	9,35	6.47	96.91	10,99	12.19
313	Sulfacetamide	0.40	0.9952	N/A	N/A	N/A	118.98	12,18	24.55	101.34	12,33	3.97	97.43	9,68	12.30	96.06	12,88	2.45
314	Sulfachloropiridacine	0.80	0.9930	N/A	N/A	N/A	N/A	N/A	N/A	93.69	13,68	3.92	102.35	8,92	8.67	94.47	14,20	0.06
315	Sulfadiacine	0.80	0.9933	N/A	N/A	N/A	N/A	N/A	N/A	97.95	12,68	8.67	95.92	10,00	14.59	98.24	12,94	6.24
316	Sulfadimetoxine	0.10	0.9962	122.34	7,01	0.77	104.34	9,54	10.58	94.70	10,65	12.38	103.45	15,28	9.68	96.93	10,87	1.88
317	Sulfadoxine	0.10	0.9949	129.86	7,48	12.83	92.91	9,62	4.59	92.41	10,95	6.06	104.11	12,45	8.47	95.40	9,22	4.00
318	Sulfameracine	0.20	0.9941	N/A	N/A	N/A	102.32	11,05	16.69	99.10	9,71	8.73	105.86	8,98	9.54	95.49	9,31	1.52
319	Sulfametacine	0.20	0.9939	N/A	N/A	N/A	98.96	12,83	1.51	97.49	11,61	3.97	103.52	8,92	8.64	95.26	13,20	1.45
320	Sulfametizole	0.80	0.9864	N/A	N/A	N/A	N/A	N/A	N/A	100.19	10,28	0.31	103.59	8,94	10.59	91.01	9,92	3.80
321	Sulfametoxazole	0.40	0.9948	N/A	N/A	N/A	112.21	12,92	9.16	94.31	14,06	6.41	103.91	10,23	10.13	94.74	14,50	3.86
322	Sulfametoxipiridacine	0.40	0.9940	N/A	N/A	N/A	104.29	13,02	13.05	98.89	13,20	19.85	105.96	12,95	6.19	95.01	14,99	3.00
323	Sulfamonomethoxine	1.20	0.9913	N/A	N/A	N/A	N/A	N/A	N/A	111.11	11,11	3.29	93.85	13,38	10.90	96.63	14,16	2.94
324	Sulfapyridine	0.40	0.9930	N/A	N/A	N/A	109.50	9,43	3.94	103.66	9,59	8.63	98.18	13,72	7.97	95.32	11,29	0.42
325	Sulfaquinoxaline	0.40	0.9962	N/A	N/A	N/A	115.47	9,35	15.86	92.67	9,83	12.76	101.15	14,97	7.60	96.86	10,75	0.16
326	Sulfatiazole	0.40	0.9922	N/A	N/A	N/A	109.32	10,34	18.92	87.79	11,88	0.71	96.90	9,58	11.07	96.24	9,72	1.08
327	Sulfisoxazole	0.80	0.9965	N/A	N/A	N/A	N/A	N/A	N/A	93.73	12,52	5.51	101.82	14,33	5.95	95.32	14,43	1.90
328	Tebuconazole	0.80	0.9949	N/A	N/A	N/A	N/A	N/A	N/A	92.08	10,26	8.58	98.28	9,74	5.09	98.61	9,22	4.53
329	Tebufenocide	0.10	0.9947	92.74	14,93	17.37	89.88	15,24	5.86	96.15	11,17	6.53	105.61	9,47	4.63	101.10	12,57	8.24
330	Tebufenpyrad	0.10	0.9950	111.56	9,92	2.79	86.18	13,25	9.05	95.86	12,29	4.97	106.62	9,27	4.29	100.57	6,66	3.07
331	Teflubenzuron	1.20	0.9860	N/A	N/A	N/A	N/A	N/A	N/A	97.01	11,66	13.94	94.86	9,78	4.53	99.61	6,65	5.63
332	Tefluthrin	0.10	0.9937	97.69	9,68	14.14	104.85	10,00	3.87	107.14	10,41	2.51	88.84	15,18	4.03	95.53	14,71	1.33
333	Telodrin (isobenzan)	0.80	0.9930	N/A	N/A	N/A	N/A	N/A	N/A	93.03	9,80	11.99	92.60	16,28	21.17	101.82	16,65	10.96
334	Terbufos	0.20	0.9942	N/A	N/A	N/A	107.98	9,37	17.24	97.29	9,28	3.06	90.31	13,69	6.42	102.47	17,11	8.75
335	Terbuthylazine	0.40	0.9961	N/A	N/A	N/A	98.38	12,63	4.02	93.37	14,53	2.45	91.01	13,07	11.41	99.87	6,61	3.75
336	Tetrachlorvinphos	0.40	0.9984	N/A	N/A	N/A	105.27	9,21	8.36	93.03	9,91	10.04	104.96	9,12	2.97	101.50	14,60	6.48
337	Tetraconazole	0.20	0.9878	N/A	N/A	N/A	96.23	12,80	9.69	101.26	13,96	5.09	89.84	14,01	3.49	99.52	6,84	8.62
338	Tetradifon	0.40	0.9912	N/A	N/A	N/A	110.62	12,25	10.72	103.75	14,09	8.41	91.10	9,45	2.57	100.16	6,91	8.47
339	Tetramethrin	1.60	0.9871	N/A	N/A	N/A	N/A	N/A	N/A	N/A	N/A	N/A	85.18	10,80	2.32	98.04	7,06	1.46
340	Thiabendazole	0.20	0.9953	N/A	N/A	N/A	99.82	9,60	4.72	88.38	9,69	3.77	101.10	9,07	5.74	99.15	18,69	4.46
341	Thiacloprid	0.20	0.9972	N/A	N/A	N/A	107.76	9,74	2.80	94.23	9,09	6.03	102.23	8,91	5.37	97.17	17,95	1.87
342	Thiamethoxam	0.80	0.9968	N/A	N/A	N/A	N/A	N/A	N/A	103.40	9,05	6.94	101.22	14,16	5.43	98.61	16,69	1.97
343	Thiophanate methyl	0.20	0.9968	N/A	N/A	N/A	102.31	13,05	6.57	94.75	16,70	4.03	103.59	14,67	7.43	97.52	7,00	3.77
344	Tolclofos methyl	0.10	0.9922	116.17	11,56	5.73	100.20	9,86	4.71	96.78	9,59	17.32	92.67	16,21	6.45	99.05	14,51	10.76
345	Tolfenamic acid	0.40	0.9917	N/A	N/A	N/A	106.53	12,83	9.72	105.64	15,03	7.99	98.88	14,27	0.15	104.07	6,78	3.23
346	Triadimefon	0.40	0.9967	N/A	N/A	N/A	115.55	12,00	19.61	95.64	14,82	2.87	100.72	9,18	4.89	99.14	6,64	0.92
347	Triadimenol	0.40	0.9949	N/A	N/A	N/A	108.82	13,57	19.22	90.50	14,42	10.27	100.86	14,76	8.82	99.02	6,64	8.06
348	Triazophos (hostathion)	0.10	0.9976	97.70	9,50	9.62	97.52	9,35	2.53	96.68	9,01	10.04	103.87	9,67	4.89	98.73	17,46	3.23
349	Trichlorfon	1.20	0.9981	N/A	N/A	N/A	N/A	N/A	N/A	99.76	9,02	19.77	100.02	8,98	0.34	98.66	16,06	0.59
350	Trifloxystrobin	0.10	0.9950	79.68	8,31	16.36	99.02	9,33	11.67	105.21	9,00	2.26	113.52	9,66	3.24	98.92	16,80	1.91
351	Triflumizole	0.10	0.9946	91.81	7,37	21.56	92.12	16,13	7.68	94.83	16,24	2.17	107.86	10,07	1.06	100.97	6,86	1.32
352	Triflumuron	0.40	0.9954	N/A	N/A	N/A	109.58	9,67	9.83	97.28	9,37	3.38	106.90	13,20	12.75	102.17	15,84	3.65
353	Trifluralin	0.20	0.9928	N/A	N/A	N/A	113.51	14,55	1.27	105.71	15,05	16.83	85.71	15,12	16.24	99.77	6,81	16.19
354	Trimethoprim	0.80	0.9974	N/A	N/A	N/A	N/A	N/A	N/A	98.59	16,81	5.49	108.01	15,11	4.37	96.85	7,03	0.59
355	Triticonazole	0.40	0.9948	N/A	N/A	N/A	119.81	11,96	4.08	97.96	15,07	9.40	99.78	14,17	1.39	98.33	6,91	1.25
356	Tylmicosin	1.60	0.9955	N/A	N/A	N/A	N/A	N/A	N/A	N/A	N/A	N/A	103.10	13,68	3.57	95.24	14,57	6.28
357	Tylosin	0.80	0.9975	N/A	N/A	N/A	N/A	N/A	N/A	105.10	9,03	6.25	103.84	14,83	4.75	99.16	18,48	5.44
358	Vinclozolin	0.20	0.9936	N/A	N/A	N/A	112.53	9,07	17.45	95.98	9,13	6.12	85.39	17,83	5.12	97.78	16,65	0.80
359	Warfarin	0.10	0.9962	106.32	9,23	20.28	90.08	13,61	15.11	92.87	16,86	4.58	103.35	13,18	3.78	98.30	6,73	0.08
360	Zoxamide	0.40	0.9960	N/A	N/A	N/A	126.95	9,61	10.37	94.83	9,07	3.13	101.49	12,65	5.65	98.29	15,52	1.08

**Fig. 1:**
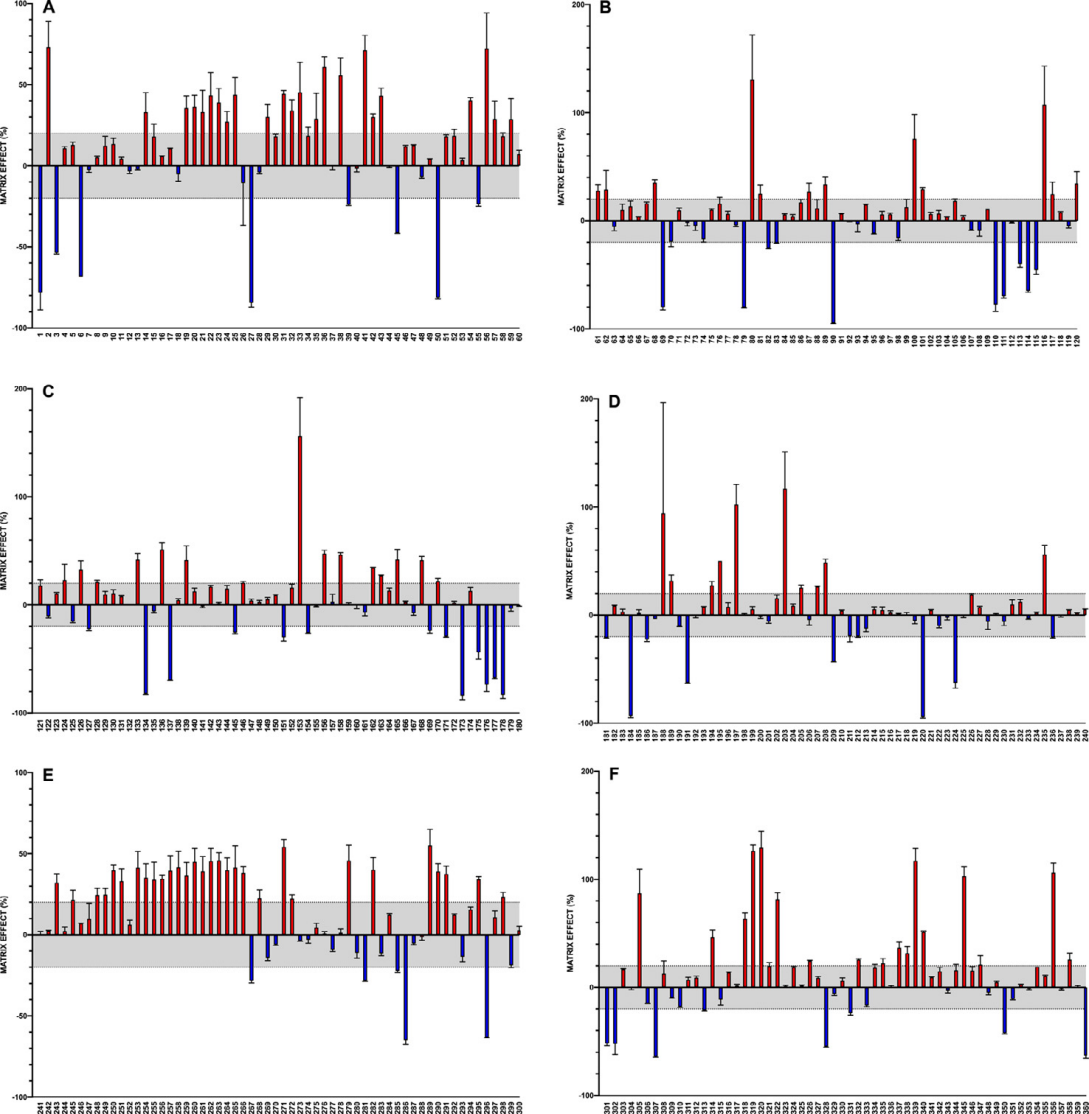
Matrix effect (%) of 360 compounds included on the method

Fig. 1 shows the effect that the components of the matrix have on the quantification of each chemical substance, to demonstrate the need to perform said quantification with calibrators prepared in a white matrix for at least 45% of the compounds. Results are shown as a relative percentage quantified by ACN calibration curve. When the differential is greater 20% or −20%, matrix interference is considered to exist. For clarity, the compounds are identified numerically, from 1 to 360, as they are numbered in Table 1. Raw data of the six charts of this figure are provided in the supplementary file 2.

Tables 2 and 3 show the quantitative results of the contaminants found in 36 barn owls (*Tyto Alba*) and Tables 4 and 5, show those found in 112 common kestrels (*Falco tinnunculus*). The results of persistent and non-persistent organic pollutants are presented separately. For clarity, only contaminants that have tested positive for at least one individual are shown. Raw data of the quantification of the 360 pollutants in these 187 birds are provided in the supplementary file 3.

**Table 2:** Concentrations of persistent organic pollutants found in whole blood (ng/ml) of a series of barn owls sampled in Castilla–Leon (Spain) during 2018 and 2019.

	**Acenaphtylene**	**BDE-47**	**BDE-100**	**BDE-153**	**BDE-154**	**BDE-99**	**Dichlorodiphenyldichloroethylene (p,p’ DDE)**	**Fluoranthene**	**Fluorene**	**Hexachlorobencene**	**PCB 118**	**PCB 138**	**PCB 153**	**PCB 156**	**PCB 167**	**PCB 180**	**PCB 189**	**Phenanthrene**	**Pyrene**
**Barn owl 01**	0.39						0.14	0.28	0.68	1.51		0.32	0.33			0.42		0.46	0.73
**Barn owl 02**		0.19	0.33	0.20	0.10	0.68	3.06		0.53	0.83	0.20	2.22	3.21	0.23	0.25	5.81	0.13	0.54	0.20
**Barn owl 03**	0.32	0.20	0.10	0.20		0.09			0.35			0.10	0.10				0.09	0.49	0.20
**Barn owl 04**							0.10		0.41			0.10	0.12				0.12	0.29	0.20
**Barn owl 05**	0.22	0.20	0.12	0.20	0.10	0.40		0.21	0.60									0.75	0.57
**Barn owl 06**							0.13		0.40	0.38		0.14	0.20				0.22	0.46	0.34
**Barn owl 07**							0.93	0.32	0.56			0.28	0.42				0.58	0.42	0.29
**Barn owl 08**																			
**Barn owl 09**							0.25		0.93	0.44		0.25	0.35				0.41	0.40	0.21
**Barn owl 10**							0.49		0.52	0.48		0.25	0.32				0.38	0.65	0.60
**Barn owl 11**							0.53	0.26	1.31	0.90	0.20	0.18	0.20				0.28	1.57	0.45
**Barn owl 12**							0.10	0.22	1.05	0.43			0.09					1.24	0.27
**Barn owl 13**									0.55	0.71								1.18	0.19
**Barn owl 14**									1.13									0.80	0.21
**Barn owl 15**	0.35								0.78	0.71								0.59	0.20
**Barn owl 16**							0.16		1.30	0.20		0.10					0.14	1.12	0.21
**Barn owl 17**	0.38								0.77	0.20								0.39	0.20
**Barn owl 18**									0.86	0.24								0.50	0.20
**Barn owl 19**									1.12	0.34								1.88	0.27
**Barn owl 20**	0.36								0.67									0.58	0.21
**Barn owl 21**								0.27	1.27									1.06	0.44
**Barn owl 22**								0.34	0.99								0.10	1.06	0.23
**Barn owl 23**								0.19	0.72									0.91	0.20
**Barn owl 24**								0.20	0.64									0.41	0.20
**Barn owl 25**							0.10	0.28	1.29	0.36							0.10	2.25	0.30
**Barn owl 26**								0.33	0.26	0.62								1.40	0.26
**Barn owl 27**							0.14	0.43	0.92	0.61								1.32	0.19
**Barn owl 28**									0.99									0.57	0.20
**Barn owl 29**							0.42		0.44	1.00		0.17	0.27				0.39	0.54	0.20
**Barn owl 30**							1.11		1.27	1.75		0.41	0.48				0.55	0.81	0.20
**Barn owl 31**									0.81	0.38								0.52	0.20
**Barn owl 32**								0.30	1.50	0.23								2.23	0.26
**Barn owl 33**									0.79	0.35								0.85	0.20
**Barn owl 34**									0.74									0.99	0.23
**Barn owl 35**									1.05	0.38								0.99	0.26
**Barn owl 36**									0.80									0.73	0.19

**Table 3:** Concentrations of non-persistent organic pollutants found in whole blood (ng/ml) of a series of barn owls sampled in Castilla–Leon (Spain) during 2018 and 2019.

	2-Phenylphenol	Benalaxyl	Brodifacoum	Bromadiolone	Coumatetralyl	Dexamethasone	Difenacoum	Enrofloxacin	Eprinomectin	Fenbendazole	Flocoumafen	Flumequine	Metaflumizone	Simazine	Sulfadiacine	Thiacloprid
**Barn owl 01**	0.20		0.80			0.40		1.20			0.20		0.14		13.69	
**Barn owl 02**	0.21	0.13	1.35	0.41												
**Barn owl 03**	0.33															
**Barn owl 04**	0.20	0.10											0.63			
**Barn owl 05**	0.24															
**Barn owl 06**	0.20															
**Barn owl 07**	0.20															
**Barn owl 08**																
**Barn owl 09**	0.36								0.31							
**Barn owl 10**	0.27												0.24			
**Barn owl 11**	5.29															
**Barn owl 12**	5.88	0.10														
**Barn owl 13**	4.19									0.10						2.57
**Barn owl 14**	1.71															
**Barn owl 15**	0.34															
**Barn owl 16**	2.60															
**Barn owl 17**	0.24															
**Barn owl 18**	0.34								0.33							
**Barn owl 19**	4.27															
**Barn owl 20**	0.29	0.20					0.40									
**Barn owl 21**	1.35	0.10														
**Barn owl 22**	0.56															0.21
**Barn owl 23**	0.32	0.09														
**Barn owl 24**	0.32													0.39		0.20
**Barn owl 25**	5.20						0.40									0.43
**Barn owl 26**	3.43															
**Barn owl 27**	2.20															
**Barn owl 28**	0.33	0.10			111.36											
**Barn owl 29**	0.38		6.37													
**Barn owl 30**	0.38															
**Barn owl 31**	0.32															
**Barn owl 32**	4.25															
**Barn owl 33**	0.41	0.15										0.10				
**Barn owl 34**	0.37															
**Barn owl 35**	0.63						0.40									
**Barn owl 36**	0.51

**Table 4:** Concentrations of persistent organic pollutants found in whole blood (ng/ml) of a series of common kestrels sampled in Castilla–Leon (Spain) during 2018 and 2019.

	**Acenaphthene**	**Acenaphtylene**	**BDE99**	**BDE100**	**BDE153**	**BDE154**	**BDE183**	$\begin{array}{c} \mathrm{Dichlorodiphenyl}\\ \mathrm{dichloroethane}(p,p^{\prime }\mathrm{DDD}) \end{array}$	$\begin{array}{c} \mathrm{Dichlorodiphenyl}\\ \mathrm{dichloroethylene}(p,p^{\prime }\mathrm{DDE}) \end{array}$	**Fluoranthene**	**Fluorene**	**Hexachlorobencene**	$\begin{array}{c} \mathrm{Hexachlorocyclohexane}\\ \left(\mathrm{alpha}\right) \end{array}$	$\begin{array}{c} \mathrm{Hexaclorocyclohexane}\\ \left(\mathrm{beta}\right) \end{array}$	**Naphtalene**	**PCB28**	**PCB101**	**PCB105**	**PCB118**	**PCB138**	**PCB153**	**PCB156**	**PCB167**	**PCB180**	**PCB189**	**Phenanthrene**	**Pyrene**
**Common kestrel 01**		0.23	0.21	0.10					4.40	0.29	0.89	0.61					0.20	0.20	0.49	1.83	2.74	0.20	0.25	0.22	0.09	1.16	0.91
**Common kestrel 02**																					0.10					0.61	0.30
**Common kestrel 03**																										0.72	0.25
**Common kestrel 04**											0.31	0.20														0.53	0.20
**Common kestrel 05**									0.62		0.40	0.36														1.26	0.20
**Common kestrel 06**		0.35							0.33		0.52									0.22	0.26			0.25		1.08	0.20
**Common kestrel 07**									0.37			0.34								0.25	0.29			0.28		0.54	0.20
**Common kestrel 08**									0.54		0.22	0.33								0.30	0.35			0.22		0.63	0.20
**Common kestrel 09**									0.21		0.28															0.44	0.20
**Common kestrel 10**																											
**Common kestrel 11**			0.10	0.10					0.41		0.56	0.27								0.17	0.33			0.37		1.19	0.20
**Common kestrel 12**											0.31	0.19														0.31	0.20
**Common kestrel 13**											0.34															0.57	0.20
**Common kestrel 14**											0.22	0.37														0.37	0.20
**Common kestrel 15**	0.20										0.40	0.34														0.55	0.20
**Common kestrel 16**											0.42	0.20														1.36	0.20
**Common kestrel 17**											0.45															0.85	0.20
**Common kestrel 18**											0.33															0.79	0.20
**Common kestrel 19**		0.35																									
**Common kestrel 20**																											
**Common kestrel 21**																											
**Common kestrel 22**											0.33	0.20												0.17		0.26	0.20
**Common kestrel 23**											0.23	0.19														0.32	0.20
**Common kestrel 24**											0.47	0.28														0.75	0.20
**Common kestrel 25**									0.10		0.55	0.20								0.10	0.09			0.16		0.79	0.20
**Common kestrel 26**									0.10			0.23								0.10	0.10			0.12		0.45	0.20
**Common kestrel 27**									0.17		0.29	0.36									0.11			0.10		1.30	0.28
**Common kestrel 28**									0.39		0.54	0.20														0.76	0.20
**Common kestrel 29**									0.10		0.36	0.47												0.09		0.57	0.20
**Common kestrel 30**									0.10		0.40	0.20												0.09		0.44	0.20
**Common kestrel 31**											0.27	0.54														0.55	0.20
**Common kestrel 32**											0.23	0.20														0.31	0.20
**Common kestrel 33**									0.10		0.20	0.20									0.10			0.11		0.30	0.20
**Common kestrel 34**		0.27									0.37	0.33														0.40	0.20
**Common kestrel 35**												0.42									0.10					0.83	0.38
**Common kestrel 36**		0.24										0.36														0.83	0.25
**Common kestrel 37**		0.20							0.09	0.25	0.25	0.39			2.67											0.57	0.23
**Common kestrel 38**									0.36	0.27	0.38	0.66														0.50	0.28
**Common kestrel 39**	0.25										0.58	0.53														1.55	0.31
**Common kestrel 40**		0.19							0.15	0.19	0.53	0.99								0.09	0.11			0.09		0.40	0.39
**Common kestrel 41**		0.28							0.66		0.63	0.95								0.09	0.15			0.10		0.96	0.43
**Common kestrel 42**		0.27								0.20	0.51	1.10														0.70	0.37
**Common kestrel 43**	0.31										1.02	0.32														1.97	0.47
**Common kestrel 44**	0.37								0.35		0.68	0.20														1.41	0.22
**Common kestrel 45**								0.65	0.91		0.57	0.32								0.10	0.10			0.10		1.01	0.21
**Common kestrel 46**		0.23									0.28	0.27														0.40	0.20
**Common kestrel 47**											0.34	0.33														0.73	0.20
**Common kestrel 48**	0.20								0.10		0.69	0.19														1.04	0.25
**Common kestrel 49**									0.10		0.31	0.20								0.10	0.09					0.86	0.21
**Common kestrel 50**									0.09		0.66	0.52												0.10		0.54	0.20
**Common kestrel 51**											0.54	0.27														0.44	0.21
**Common kestrel 52**									0.10		0.42	0.44														0.91	0.21
**Common kestrel 53**											0.51	0.78														0.45	0.20
**Common kestrel 54**											0.35	0.58														0.81	0.20
**Common kestrel 55**									0.38	0.38	0.48	1.14								0.09	0.19			0.19		1.27	0.24
**Common kestrel 56**									0.29		0.41	0.75								0.10	0.19			0.20		0.76	0.20
**Common kestrel 57**									0.83		0.64	1.02								0.17	0.33			0.35		0.53	0.25
**Common kestrel 58**											0.46															0.70	0.25
**Common kestrel 59**											0.65															0.57	0.22
**Common kestrel 60**											0.54															0.21	0.20
**Common kestrel 61**											0.35	0.36														0.35	0.20
**Common kestrel 62**											0.45	0.17														0.35	0.20
**Common kestrel 63**									0.32	0.23	0.38								0.20	0.13	0.36			0.44		0.51	0.70
**Common kestrel 64**					0.20				0.10		0.45	2.51	0.40	5.95					0.20	0.64	3.49	0.23	0.27	6.88	0.18	0.52	0.28
**Common kestrel 65**		0.45							1.40		0.59	5.49							0.26	0.82	2.83	0.20	0.19	2.80		1.45	0.29
**Common kestrel 66**									0.12		0.63	2.86								0.14	0.23			0.26		0.86	0.20
**Common kestrel 67**									0.11		0.41	2.72								0.18	0.49		0.10	0.69		0.85	0.20
**Common kestrel 68**									0.10		0.39	0.38								0.10	0.11			0.11		0.71	0.20
**Common kestrel 69**											0.48	0.39								0.10	0.20			0.27		0.62	0.20
**Common kestrel 70**											0.51	0.49														1.08	0.20
**Common kestrel 71**									2.03		0.32	2.63				0.10			0.20	0.24	0.83		0.09	0.90		0.38	0.20
**Common kestrel 72**										0.36	0.47	0.24														1.22	0.22
**Common kestrel 73**											0.31	0.31														0.38	0.20
**Common kestrel 74**											0.46	0.20														1.15	0.20
**Common kestrel 75**		0.35									0.46	0.38														0.39	0.20
**Common kestrel 76**											0.36															0.56	0.20
**Common kestrel 77**									0.10		0.57	0.56									0.10					0.73	0.24
**Common kestrel 78**									0.09		0.40									0.10	0.10			0.13		0.39	0.20
**Common kestrel 79**											0.45	0.39														0.74	0.20
**Common kestrel 80**											0.45	0.27														0.35	0.20
**Common kestrel 81**										0.36	0.47															0.79	0.25
**Common kestrel 82**											0.46	0.28														0.42	0.20
**Common kestrel 83**										0.26	0.41															0.46	0.34
**Common kestrel 84**											0.81	0.30														1.04	0.23
**Common kestrel 85**											0.61	0.35														0.56	0.21
**Common kestrel 86**											0.54	0.25														0.52	0.20
**Common kestrel 87**										0.24	0.55															0.99	0.59
**Common kestrel 88**										0.25	0.83															1.65	0.30
**Common kestrel 89**									0.09		0.46									0.13	0.26			0.26		0.33	0.21
**Common kestrel 90**									0.37		0.36								0.20	0.53	0.98	0.20	0.12	0.90		0.45	0.20
**Common kestrel 91**			0.23	0.12	0.20	0.10	0.20		4.29		0.42							0.17	0.50	2.87	5.17	0.49	0.36	6.80		1.05	0.25
**Common kestrel 92**											0.50	0.57														0.53	0.21
**Common kestrel 93**		0.21									0.42	0.49														0.44	0.20
**Common kestrel 94**											0.59	0.73									0.11			0.13		0.55	0.20
**Common kestrel 95**			0.10						0.11	0.29	0.59										0.10			0.10		1.04	0.36
**Common kestrel 96**											0.22	0.26								0.10	0.10			0.10		0.59	0.20
**Common kestrel 97**										0.26	0.51	0.53			0.81									0.10		0.65	0.22
**Common kestrel 98**									0.37		0.39	0.48			0.83					0.10	0.14			0.11		0.97	0.21
**Common kestrel 99**		0.37							0.15		0.35	0.44								0.13	0.19			0.24		0.98	0.21
**Common kestrel 100**		0.42									0.35															0.88	0.30
**Common kestrel 101**		0.44							0.31		0.40	0.41														0.34	0.20
**Common kestrel 102**											0.46															1.05	0.20
**Common kestrel 103**	0.37									0.21	0.23															0.47	0.20
**Common kestrel 104**		0.31							0.09	0.25	0.41															0.40	0.40
**Common kestrel 105**	0.29										0.33															0.51	0.27
**Common kestrel 106**																											
**Common kestrel 107**			0.09	0.10					0.18		0.39	0.29								0.20	0.26			0.62		0.39	0.20
**Common kestrel 108**		0.40	0.10						1.44		0.30	0.83								0.41	0.61			0.70		0.37	0.20
**Common kestrel 109**		0.42							0.22		0.43	0.51								0.10	0.24			0.36		0.25	0.20
**Common kestrel 110**											0.81	0.50							0.20	0.16	0.46			0.70		0.45	0.20
**Common kestrel 111**	0.51								0.10		0.57															0.79	0.20
**Common kestrel 112**		0.26									0.38	1.27								0.10	0.21			0.38		0.45	0.20

**Table 5:** Concentrations of non-persistent organic pollutants in whole blood (ng/ml) of a series of common kestrels sampled in Castilla–Leon (Spain) during 2018 and 2019.

	**2-Phenylphenol**	**Albendazole**	**Atrazine**	**Benalaxyl**	**Brodifacoum**	**Bromadiolone**	**Coumachlor**	**Coumaphos**	**Coumatetralyl**	**Difenacoum**	**Difethialone**	**Diphenylamine**	**Enrofloxacin**	**Fenbendazole**	**Flumequine**	**Levamisole**	**Mebendazole**	**Metaflumizone**	**Metrafenone**	**Simazine**	**Sulfachloropiridacine**	**Sulfadiacine**	**Sulfapyridine**
**Common kestrel 01**	0.20																						
**Common kestrel 02**	1.23															0.52							
**Common kestrel 03**	2.25			0.10																			
**Common kestrel 04**	0.81															0.42							
**Common kestrel 05**	1.31			0.10								0.51											
**Common kestrel 06**	0.87											0.23						0.20					
**Common kestrel 07**	0.52											0.25				0.31		0.20					
**Common kestrel 08**	2.05											0.31				0.28		0.20					
**Common kestrel 09**	0.58											0.21				0.33							
**Common kestrel 10**																		0.30	0.10				
**Common kestrel 11**	1.73				0.80													0.37					
**Common kestrel 12**	0.37															0.28							
**Common kestrel 13**	0.59							0.11				0.28											
**Common kestrel 14**	0.67															0.21		0.19					
**Common kestrel 15**	0.73			0.10			5.84																
**Common kestrel 16**	5.27											0.63		0.10		0.22							
**Common kestrel 17**	3.90																						
**Common kestrel 18**	0.98	0.10										0.20											
**Common kestrel 19**																							
**Common kestrel 20**				0.10												0.24							
**Common kestrel 21**				0.17																			
**Common kestrel 22**	0.64							0.19															
**Common kestrel 23**	1.07															0.21							
**Common kestrel 24**	1.09																						
**Common kestrel 25**	1.20				0.80																		
**Common kestrel 26**	0.45				0.80																		
**Common kestrel 27**	1.04																						
**Common kestrel 28**	0.99																	0.58					
**Common kestrel 29**	0.52					0.40												0.19					
**Common kestrel 30**	1.55			0.10											0.10			0.20					
**Common kestrel 31**	0.78																						
**Common kestrel 32**	1.07			0.10																			
**Common kestrel 33**	0.82															0.23							
**Common kestrel 34**	0.41																	0.92					
**Common kestrel 35**	1.14											0.20											
**Common kestrel 36**	0.60																						
**Common kestrel 37**	0.36																		0.10				
**Common kestrel 38**	1.11																						
**Common kestrel 39**	4.91			0.10								0.50											
**Common kestrel 40**	0.84																						
**Common kestrel 41**	1.55																						
**Common kestrel 42**	0.60																						
**Common kestrel 43**	2.35											0.33											
**Common kestrel 44**	2.21				0.98							0.65											
**Common kestrel 45**	1.80				0.80																		
**Common kestrel 46**	0.21			0.10																			
**Common kestrel 47**	0.32																						
**Common kestrel 48**	2.65			0.10								0.20											
**Common kestrel 49**	0.91								13.02														
**Common kestrel 50**	0.34																						
**Common kestrel 51**	0.60				0.80																		
**Common kestrel 52**	0.48																						
**Common kestrel 53**	0.32			0.10																			
**Common kestrel 54**	0.26			0.10																			
**Common kestrel 55**	1.56																						
**Common kestrel 56**	0.29																						
**Common kestrel 57**	0.20																						
**Common kestrel 58**	0.21																						
**Common kestrel 59**	0.60																						
**Common kestrel 60**	2.01																						
**Common kestrel 61**	0.70																						
**Common kestrel 62**	0.78																						
**Common kestrel 63**	0.21									0.41			1.20										
**Common kestrel 64**	0.19																0.25						
**Common kestrel 65**	0.24															0.25							
**Common kestrel 66**	1.45																						
**Common kestrel 67**	1.09																						
**Common kestrel 68**	0.75			0.10																			
**Common kestrel 69**	1.00																						
**Common kestrel 70**	0.54																						
**Common kestrel 71**	0.99				32.73					0.42											0.80		
**Common kestrel 72**	0.78																						
**Common kestrel 73**	0.68																						
**Common kestrel 74**	2.50																						
**Common kestrel 75**	0.20																						
**Common kestrel 76**	0.66																						
**Common kestrel 77**	0.59			0.16																0.22			
**Common kestrel 78**	0.20			0.23	1.93				0.30		1.77											0.85	
**Common kestrel 79**	1.32			0.20														0.20	0.10	0.29			
**Common kestrel 80**	0.20		0.1																	0.24			
**Common kestrel 81**	1.20			0.26																0.23			
**Common kestrel 82**	0.20			0.14																			0.40
**Common kestrel 83**	0.20			0.14															0.10				
**Common kestrel 84**	1.98			0.16																0.19			
**Common kestrel 85**	0.37			0.16										0.10									
**Common kestrel 86**	0.29			0.10										0.10									
**Common kestrel 87**	0.32											0.46											
**Common kestrel 88**	1.32			0.10																			
**Common kestrel 89**	0.30																						
**Common kestrel 90**	0.20																						
**Common kestrel 91**	2.99																						
**Common kestrel 92**	0.20																			0.20			
**Common kestrel 93**	0.20			0.10																			
**Common kestrel 94**	0.20																						
**Common kestrel 95**	0.39																						
**Common kestrel 96**	0.20																						
**Common kestrel 97**	0.43																	0.20					
**Common kestrel 98**	0.26			0.10														0.24					
**Common kestrel 99**	0.20			0.10																			
**Common kestrel 100**	0.51											0.20											
**Common kestrel 101**	0.29																						
**Common kestrel 102**	0.47											0.49											
**Common kestrel 103**	0.60																						
**Common kestrel 104**	0.52																						
**Common kestrel 105**	0.42																						
**Common kestrel 106**																							
**Common kestrel 107**	0.20																						
**Common kestrel 108**	0.20																						
**Common kestrel 109**	0.45																						
**Common kestrel 110**	0.80					0.42																	
Common kestrel 111	2.09											0.82											
**Common kestrel 112**	0.24																						

## Experimental design, materials, and methods

### Chemicals, reagents, and calibrators

Certified pure standards of the 360 chemicals included in the methodology (Dr Ehrestorfer, Augsburg, Germany; CPA Chem, Stara Zagora, Bulgaria; A2S – Analytical Standard Solutions, Staint Jean D'Illac, France; Sigma-Aldrich, Augsburg, Germany; Accustandard, New Haven, USA; and European Pharmacopoeia Reference Standards, Strasbourg, France) were individually prepared at 1 mg/ml in a suitable solvent. From those, three intermediate working solutions (by groups: pesticides, medicaments and COPs) containing all the analytes at 1 μg/ml was prepared. This working solution was employed to prepare 12-point calibration curve, either in 1% FA-ACN or in whole blood (a 1:1 mixture of chicken and goat blood, obtained from healthy animals in the Veterinary Faculty of the University of Las Palmas de Gran Canaria). The intermediate solution was also employed for preparing quality controls (QC) and all the fortification levels in blank matrix and extracted blank matrix, that were employed in the validation experiments. The salts for the QuEChERS extraction according to the AOAC method were acquired in commercial premixes from Agilent Technologies (Palo Alto, USA). Acetonitrile (ACN), methanol (MeOH), and FA were of the maximum purity available and were purchased from Honeywell (Charlotte, USA). Ammonium acetate was from Fisher (Fisher Scientific UK, Loughborough, UK). The water was prepared in the laboratory using an ultra purification system (Millipore, Molsheim, France).

### Sample preparation

Two hundred fifty microliters whole blood from wild birds or blank matrix (goat + chicken whole blood) either fortified with the 360 analytes plus the internal standards (ISs), the ISs alone, or without fortification, were subjected to an AOAC-QuEChERS extraction method (one-step, without cleanup). The mixture of IS (acenaphthene-d10, chlorpyrifos-d10, chrysene-d12, diazinon-d10, PCB 200, and phenanthrene-d10 for the GC method, atrazine-d5, carbendazim-d3, cyromazine-d4, diazinon-d10, linuron-d3, and pirimicarb-d6 for the LC method) was added to all the tubes before the extraction. The samples were orbital shaken for 1 h, and then, 500 µl of 1% FA-ACN were added, before tubes were placed in an ultrasonic bath at room temperature for 20 min. Then, the QuEChERS salts (150 mg anhydrous magnesium sulfate and 37.5 mg sodium acetate) were added and, the tubes were vortexed 30 s and vigorously manually shaken for 60 s. After a 5 min centrifugation, the supernatant was filtered (0.2 µm) and used directly for chromatographic analyses. The optimization of the procedure is detailed in the main article [3].

### Chromatographic analyses

Two complementary analyses were needed for the quantification of 360 environmental pollutants in whole blood. Thus, 234 compounds were analyzed by liquid chromatography and other 126 chemicals were analyzed by gas chromatography. Both techniques were tandem coupled to triple quadrupole mass spectrometry (LC-MS/MS and GC-MS/MS). All the equipment employed was from Agilent Technologies (Palo Alto, USA): UHPLC 1290 coupled to 6460 mass spectrometer for LC, and a 7890B GC coupled to 7010 mass spectrometer for GC. The chromatographic separations in LC were performed using an InfinityLab Poroshell 120 (2.1 mm × 100 mm, 2.7 µm). The stationary phase in GC consisted of two fused silica ultra-inert capillary columns Agilent HP-5MS (15 m × 0.25 mm i.d., 0.25 µm film thickness), that were connected by a purged union, to allow the backflushing. All the conditions in which these apparatus were operated, as well as the optimization procedure, are described in detail in the main article [3].

### Validation

This article provides supporting information on the data of the validation process, which was carried out according to the criteria established in the SANTE and SWGTOX guide [1, 2] and, taking into account our previous experience in developing and validating chromatographic methods in complex biological matrices [4], [5], [6]. Although initially the experiments were carried out with chicken and goat blood separately, we found no significant differences. Therefore, we decided to use a 1: 1 mixture of both blood types to complete all validation experiments, following the recommendations of the SWGTOX guide. All the parameters of the validation process are shown in this article, except for the identity and selectivity parameters, which are shown in Table 1 of the main article [3], and carryover, which is also explained in the main article. As the blank matrix was not completely free of all of the contaminants, the signal of the blanks was subtracted from fortified samples in all the experiments.

Linearity was assessed within the range of concentrations that were considered appropriate for the purpose of biomonitoring (0.1 to 20 ng/ml). All the calibrators (12 points) were individually prepared in blank matrix in quintuplicate by adding an appropriate volume of fortification solution. Only compounds that showed a correlation coefficient (*R*^2^) higher than 0.93 were kept in the method.

The accuracy (bias and precision) was calculated for all the 12 fortification levels injected in the chromatographic systems in quintuplicate. The bias is expressed as the percentage of the theoretical level of fortification. As recommended in the guidelines, only compounds with recoveries between 70% and 120% were kept in the method. However, due to their importance for biomonitoring certain exceptions were admitted, including some compounds with recovery percentages below or above these limits, but which were highly reproducible. Precision (repeatability and reproducibility) is expressed in terms of relative standard deviation (RSD) of the different replicates. For the reproducibility of the method, only 5 levels of fortification (0.1, 0.5, 1, 5, and 20 ng/ml) were prepared in triplicate on three different days, within a period of 2 weeks. Therefore, the RSDs were calculated from 9 values. Only those compounds in which the RSD values were less than 20% remained in the method. In Table 1, we show the data of these 3 parameters only for the five levels of fortification mentioned above, although the rest also met the specified criteria.

The LOQ of this methodology was calculated over five runs of fortified blank matrix samples of three different sources (chicken, goat, and a mixture of both), as recommended (Scientific Working Group for Forensic, 2013). The lowest non-zero calibrator approximation was employed to calculate de LOQs. This means that the lowest point of the calibration curve that complied identity, bias and precision criteria was set as the LOQ for a given compound. All compounds with LOQ> 5 ng/ml were eliminated from the method since these levels are not considered adequate for biomonitoring studies. The calculated LOQs are shown in Table 1.

The influence of the matrix components on the performance of the method was evaluated by applying the extraction method to a sufficient quantity of blank whole blood to produce a blank matrix extract, which was subsequently fortified at three levels for the mixture of 360 chemicals (0.2, 2, and 20 ng/ml), and quantified against a calibration curve prepared in the solvent (1% FA-acetonitrile). The data corresponding to these experiments are shown in Fig. 1.

### Application of the methodology to a series of blood samples of wild birds

The validated methodology was applied to a series of 148 real samples, composed of 36 samples from barn owls (*Tyto alba*) and 112 samples from common kestrels (*Falco tinnunculus*). These raptors were chosen as representative species of nocturnal and diurnal raptors and were sampled in the context of a project aimed to verify the penetration of anticoagulant rodenticides into the trophic chain of these species. Nest boxes located in the provinces of Palencia, Salamanca, Burgos, Segovia, Valladolid, and Zamora (Castilla-León, Spain) were sampled after a campaign with rodenticides against a common vole (*Microtus arvalis*) plague. All samples were collected after obtaining the corresponding permits and following the animal welfare protocols during the sampling [7]. The obtained data are shown in Tables 2 and 3 (barn owls) and Tables 3 and 4 (common kestrels).

## Declaration of Competing Interest

The authors declare that they have no known competing financial interests or personal relationships which have, or could be perceived to have, influenced the work reported in this article.

## Appendix Supplementary materials

Image, application 1

Image, application 2

Image, application 3

## References

[1] EC *SANTE/12682/2019. Guidance Document on Analytical Quality Control and Method Validation Procedures for Pesticide Residues and Analysis in Food and Feed*. 2019.

[2] SWGTOX (2013). Scientific Working Group for Forensic Toxicology (SWGTOX) standard practices for method validation in forensic toxicology. J. Anal. Toxicol..

[3] Rial-Berriel C. (2020). Micro QuEChERS-based method for the simultaneous biomonitoring in whole blood of 360 toxicologically relevant pollutants for wildlife. Sci. Total Environ..

[4] Luzardo O.P. (2013). Multi-residue method for the determination of 57 persistent organic pollutants in human milk and colostrum using a QuEChERS-based extraction procedure. Anal. Bioanal. Chem..

[5] Luzardo O.P., Ruiz-Suárez N, Valerón PF (2014). Methodology for the identification of 117 pesticides commonly involved in the poisoning of wildlife using GC-MS-MS and LC-MS-MS. J. Anal. Toxicol..

[6] Luzardo O.P. (2015). Validated analytical methodology for the simultaneous determination of a wide range of pesticides in human blood using GC-MS/MS and LC-ESI/MS/MS and its application in two poisoning cases. Sci. Justice.

[7] Espin S. (2016). Tracking pan-continental trends in environmental contamination using sentinel raptors-what types of samples should we use?. Ecotoxicology.

